# NOD1 mediates interleukin-18 processing in epithelial cells responding to *Helicobacter pylori* infection in mice

**DOI:** 10.1038/s41467-023-39487-1

**Published:** 2023-06-26

**Authors:** L. S. Tran, L. Ying, K. D’Costa, G. Wray-McCann, G. Kerr, L. Le, C. C. Allison, J. Ferrand, H. Chaudhry, J. Emery, A. De Paoli, N. Colon, S. Creed, M. Kaparakis-Liaskos, J. Como, J. K. Dowling, P. A. Johanesen, T. A. Kufer, J. S. Pedersen, A. Mansell, D. J. Philpott, K. D. Elgass, H. E. Abud, U. Nachbur, B. A. Croker, S. L. Masters, R. L. Ferrero

**Affiliations:** 1 Centre for Innate Immunity and Infectious Diseases, Hudson Institute of Medical Research, Melbourne, VIC Australia; 2 Department of Molecular and Translational Science, Monash University, Melbourne, VIC Australia; 3 Department of Anatomy and Developmental Biology, Development and Stem Cells Program, Biomedicine Discovery Institute, Monash University, Melbourne, VIC Australia; 4 Department of Microbiology, Biomedicine Discovery Institute, Monash University, Melbourne, VIC Australia; 5 Monash Micro Imaging, Monash University, Melbourne, VIC Australia; 6 Department of Immunology, University of Hohenheim, Institute of Nutritional Medicine, Stuttgart, Germany; 7 TissuPath, Melbourne, VIC Australia; 8 Department of Immunology, University of Toronto, Toronto, ON Canada; 9 Cell Signalling and Cell Death Division, WEHI, Melbourne, VIC Australia; 10 Boston Children’s Hospital, Harvard Medical School, Boston, MA USA; 11 Inflammation Division, WEHI, Melbourne, VIC Australia

**Keywords:** Bacterial pathogenesis, Antimicrobial responses, Interleukins, Mucosal immunology

## Abstract

The interleukin-1 family members, IL-1β and IL-18, are processed into their biologically active forms by multi-protein complexes, known as inflammasomes. Although the inflammasome pathways that mediate IL-1β processing in myeloid cells have been defined, those involved in IL-18 processing, particularly in non-myeloid cells, are still not well understood. Here we report that the host defence molecule NOD1 regulates IL-18 processing in mouse epithelial cells in response to the mucosal pathogen, *Helicobacter pylori*. Specifically, NOD1 in epithelial cells mediates IL-18 processing and maturation via interactions with caspase-1, instead of the canonical inflammasome pathway involving RIPK2, NF-κB, NLRP3 and ASC. NOD1 activation and IL-18 then help maintain epithelial homoeostasis to mediate protection against pre-neoplastic changes induced by gastric *H. pylori* infection in vivo. Our findings thus demonstrate a function for NOD1 in epithelial cell production of bioactive IL-18 and protection against *H. pylori*-induced pathology.

## Abstract (web summary)

Maturation of interleukin-18 (IL-18) is normally mediated by activated inflammasomes in innate immune cells as an antipathogen mechanism. Here the authors show that IL-18 maturation occurs in gut epithelial cells upon encounter with *Helicobacter pylori*via a noncanonical NOD1/caspase-1 pathway to maintain epithelial homoeostasis.

## Introduction

Interleukin-1 (IL-1) family cytokines (e.g. IL-1α, IL-1β, IL-18) play important roles in inflammatory disorders of the gastrointestinal tract^1^. These cytokines undergo proteolytic cleavage to become biologically active^1^. The processing of IL-1 family members IL-1β and IL-18 is mediated by multi-protein complexes, known as “inflammasomes”, which can play both beneficial and deleterious roles in the host during infection^2,3^. Five proteins were reported to form “canonical inflammasomes”, with several others awaiting confirmation^2^. The confirmed proteins are nucleotide-binding domain (NBD) and leucine-rich repeat (LRR)-containing (NLR) protein containing a pyrin domain 1 (NLRP1); NLRP3; NLR caspase activation and recruitment domain-containing protein 4 (NLRC4); absent in melanoma 2 (AIM2); and pyrin. These proteins act as sensors, forming molecular scaffolds for the recruitment of pro-caspase-1, leading to cytokine processing and induction of inflammatory cell death^2^.

According to the current model of canonical inflammasome formation, sensor proteins with a pyrin domain (PYD) respond to cellular signals by forming oligomeric structures, then bind via PYD–PYD interactions with the adaptor protein, apoptosis-associated speck-like protein (ASC)^2^. In turn, ASC interacts with pro-caspase-1 through homotypic caspase-activation recruitment domain (CARD) binding, resulting in cleavage of pro-caspase-1 and subsequent processing of IL-1β/IL-18 into their biologically active forms^2^. Alternatively, inflammasome sensor proteins may bind with caspase-1 via CARD–CARD interactions to initiate cytokine processing (e.g. NLRC4)^2^. These models of canonical inflammasome activation have been developed from studies on IL-1β responses in haematopoietic cells, particularly those of the myeloid cell lineage. In contrast, knowledge regarding inflammasome functions in non-haematopoietic cell lineages, such as epithelial cells, is much less well understood^3^. It was suggested that inflammasomes in epithelial cells are more likely to be directed at IL-18 and not IL-1β processing^3^.

*IL18* is expressed constitutively in the gastrointestinal tract and is upregulated in response to infection by the gastric mucosal pathogen, *Helicobacter pylori*^4–6^. The role of IL-18 in gastric *H. pylori* infection is currently unclear, with both pro-inflammatory^4–6^ and anti-inflammatory^7^ activities having been assigned to this cytokine. *H. pylori* generally causes chronic gastritis, but in ~1% of cases, infection leads to gastric cancer^8^. According to the “Correa model” of gastric carcinogenesis, there is a histological progression from chronically inflamed stomach mucosa to increasingly severe changes in the glandular epithelium, characterised by atrophic gastritis, intestinal metaplasia and dysplasia, resulting finally in invasive carcinoma^9^. Several of these pre-neoplastic changes are observed in mouse gastric cancer models; however, these changes are more reflective of repair and regeneration than of carcinogenesis^10^.

*H. pylori* strains that harbour a type IV secretion system (T4SS) are more frequently associated with gastric carcinogenesis than strains lacking this factor^11^. The *H. pylori* T4SS, which is encoded by the cag pathogenicity island (*cag*PAI), is essential for *H. pylori* induction of nuclear factor-κB (NF-κB)-dependent pro-inflammatory responses in human epithelial cells^12^. It is now clear that *H. pylori* strain harbouring a functional T4SS can activate NF-κB via two innate immune signalling pathways^12^. One of these pathways involves sensing by the NLR family member nucleotide-binding oligomerisation domain 1 (NOD1), which is expressed in gastric epithelial cells and responds to cell wall peptidoglycan fragments (muropeptides) delivered by the T4SS^13^. It was also shown that the inactivation of one or more of the ~30 *cag* genes in the *cag*PAI, such as *cagM*, resulted in a non-functional T4SS and abrogated NOD1 responses in cells^13^. Importantly, *H. pylori* and other Gram-negative bacteria can also activate the NOD1 signalling pathway independently of the T4SS via the release of membrane vesicles (MVs) that carry muropeptides into the cytosol of host cells^14,15^. Upon activation, NOD1 undergoes self-oligomerisation, leading to the exposure of its CARD and recruitment of the scaffolding kinase protein, receptor-interacting serine-threonine kinase 2 (RIPK2)^16^. This leads to the activation of NF-κB and mitogen-activated protein kinase (MAPK) cascades, resulting in cytokine and chemokine production^13,14,17,18^. More recently, researchers identified a second signalling pathway, independent of NOD1, that mediates *H. pylori*-induced inflammatory responses in gastric epithelial cells involving alpha-kinase 1 (ALPK1) and tumour necrosis factor (TNF) receptor-associated factor-interacting protein with a forkhead-associated domain (TIFA) (reviewed in^12^).

Here, we identify a new role for NOD1 in mediating epithelial cell production of bioactive IL-18 in response to *H. pylori* infection and show that this occurs independently of canonical inflammasome pathways, involving instead direct interactions between NOD1 and caspase-1. We show that the NOD1-mediated formation of bioactive IL-18 prevents excessive pathology induced by chronic *H. pylori* infection in mouse models. NOD1 maintains tissue homoeostasis through the regulation of epithelial cell proliferation and apoptosis. We suggest that NOD1 may play a similar homoeostatic role in other mucosal infections. These findings highlight the importance of considering inflammasome functions in a cell-specific context that is relevant to the specific infection or disease.

## Results

### Epithelial cells are a major source of IL-18 in the gastric mucosa

IL-18 is expressed by many different tissues throughout the body, including the gastrointestinal tract, where it is produced by epithelial cells, macrophages and dendritic cells^1,19^. In the stomach, *IL18* expression is constitutive but is upregulated in response to infection by the pathogenic bacterium, *H. pylori*^4–7^. Given this observation, together with the fact that epithelial cells are a major target cell population for *H. pylori*–host interactions^12^, we sought to investigate inflammasome-mediated processing of IL-18 in epithelial cell responses to *H. pylori* infection. It was first necessary, however, to demonstrate epithelial cells as a source of IL-18 in the stomach.

**Fig. 1:**
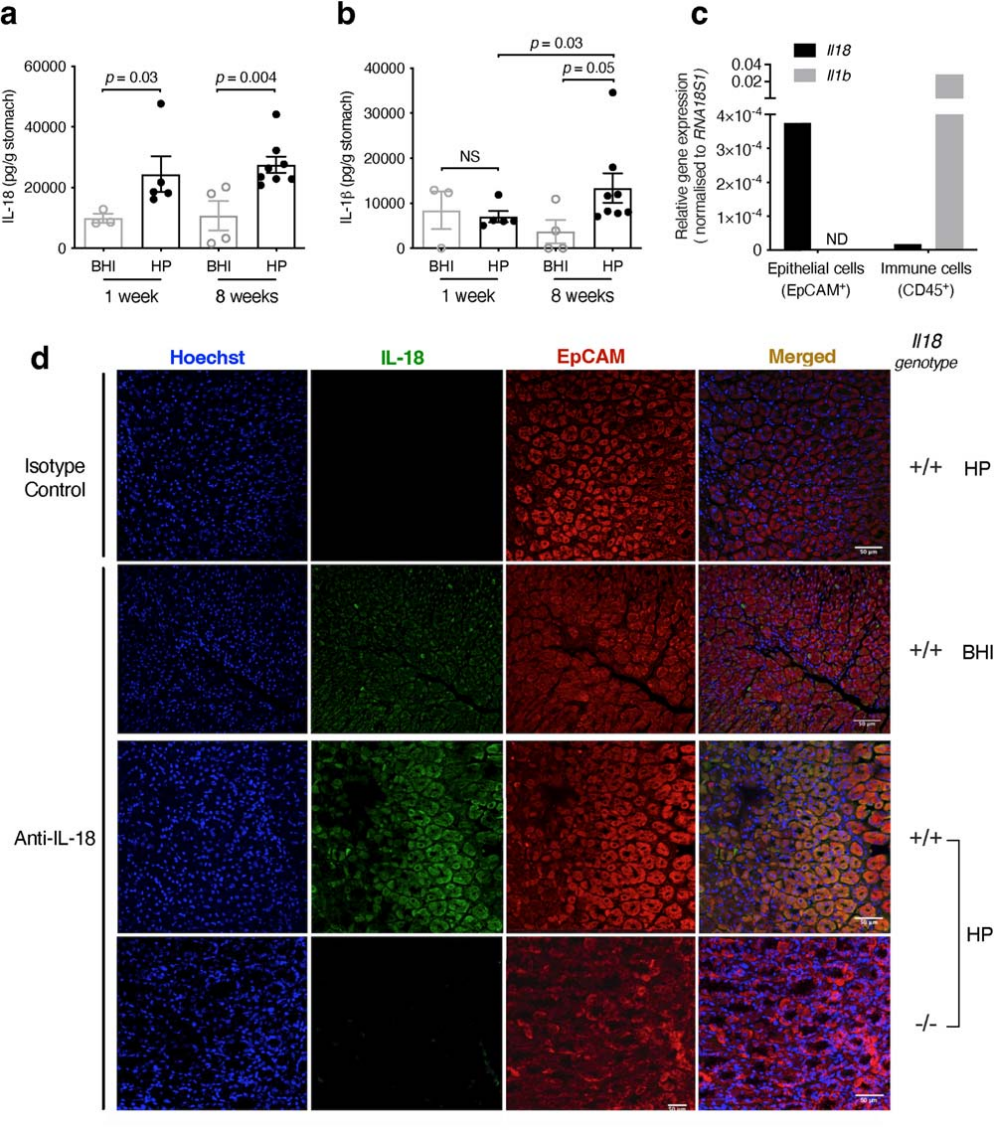
Epithelial cells are a major source of gastric IL-18 in response to *H. pylori* infection. **a** IL-18 and **b** IL-1β levels in stomach homogenates from mice administered either BHI broth (BHI) or *H. pylori* SS1 (HP) at 1 and 8 weeks p.i. (*n* = 8 and 12 male mice, respectively) **c** Epithelial (EpCAM^+^ CD45^−^) and immune (CD45.2^+^ EpCAM^−^) cells were isolated by FACS from the gastric tissues of *H. pylori*-infected mice and tested for *Il18* and *Il1b* expression by qPCR. **d** Gastric tissue sections from *H. pylori*-infected *Il18*^+/+^ and *Il18*^−/−^ mice were reacted with rat anti-mouse IL-18 and rabbit anti-EpCAM antibodies, followed by anti-rat Alexa Fluor® 488- and anti-rabbit Alexa Fluor® 594 conjugated secondary antibodies. Mouse IgG was used as an isotype control for IL-18 staining. Cell nuclei were stained with Hoechst 33342 and the sections analysed by confocal microscopy. Representative images from two independent experiments. Scale bar = 50 µm. **a**, **b** Data combined from two independent experiments. Each data point represents an individual mouse. Data correspond to the mean ± SEM. Significance was determined by a two-sided Student’s *t*-test. ND not detected.

For this, we infected mice with the mouse-adapted *H. pylori* SS1 strain and then assessed the *Il18* expression levels of the gastric cell populations isolated from these animals by fluorescence-activated cell sorting (FACS) at 1- and 8-weeks post-infection (p.i.), representative of acute and chronic infection, respectively. As a comparison, we also measured *Il1b* expression, because it has a very different expression profile in tissues to that of *IL18*^19^. Consistent with studies in mice and humans^4–7^, we detected constitutive IL-18 production in mice, whereas its production was significantly increased in animals at both 1- and 8-weeks p.i. when compared with control animals given brain heart infusion (BHI) broth alone (Fig. 1a; *p* = 0.03 and *p* = 0.004, respectively). In contrast, gastric IL-1β production was only increased at 8 weeks p.i. (Fig. 1b; *p* = 0.05). FACS analyses of isolated gastric mucosal cells from *H. pylori*-infected mice demonstrated that *Il18* gene expression levels in gastric epithelial cells (EpCAM^+^ CD45^−^) were 22-fold higher than those of the immune cell (EpCAM^−^ CD45^+^) population, whereas *Il1b* was highly expressed in immune cells but undetectable in epithelial cells (Fig. 1c). Immunofluorescence analysis further confirmed that EpCAM^+^ cells represent an important source of IL-18 in the gastric mucosa (Fig. 1d). Hence, although both IL-18 and IL-1β production is induced in the gastric mucosa during *H. pylori* infection, these cytokines display distinct cellular sources of origin, with IL-18 predominantly originating from epithelial cells.

### Non-haematopoietic cells produce IL-18, which protects against pre-neoplastic lesions during chronic *H. pylori* infection

**Fig. 2:**
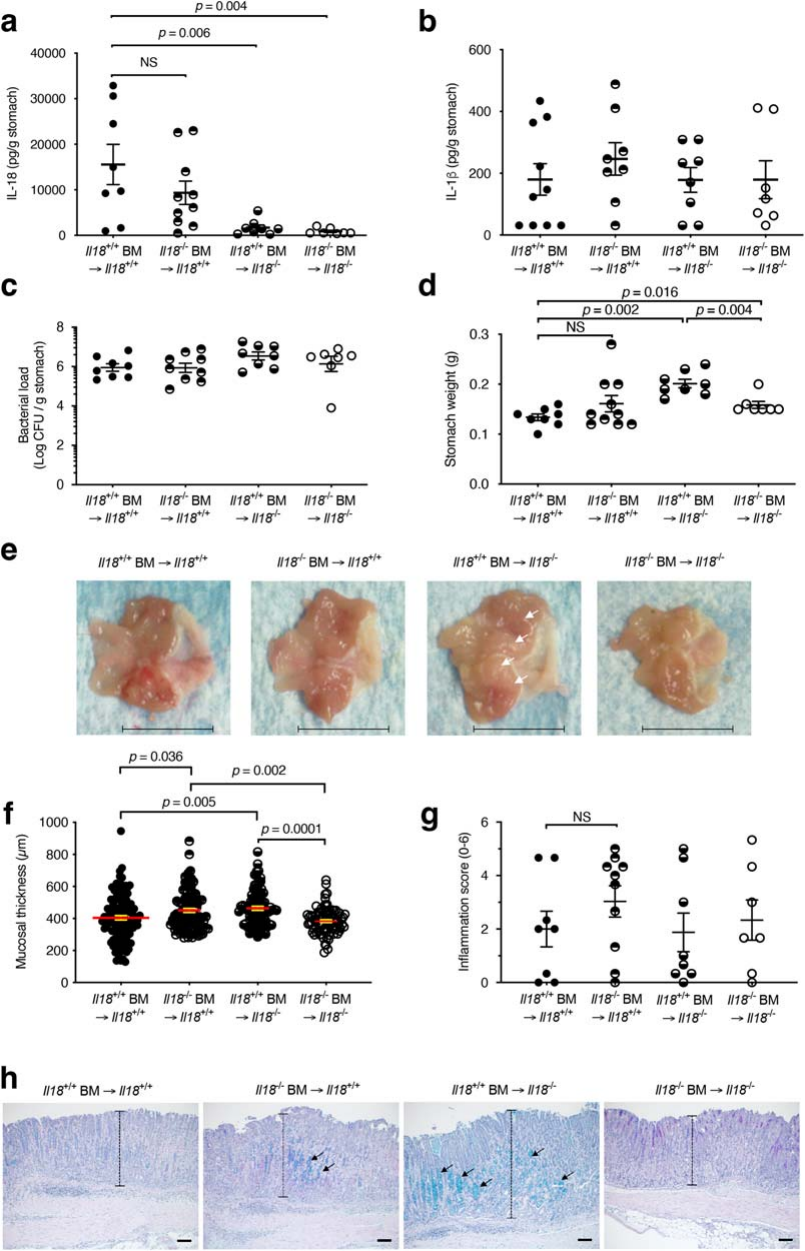
Non-haematopoietic cells produce IL-18, which protects against pre-neoplastic lesions in *H. pylori* infection. BM reconstitution experiments were performed by transferring BM from either *Il18*^+/+^ or *Il18*^−/−^ donor mice to γ-irradiated *Il18*^+/+^ or *Il18*^−/−^ recipient mice (*n*  = 33; 21 males, 12 females). Mice were then challenged with *H. pylori* SS1 and culled at 5 weeks p.i. **a** IL-18 and **b** IL-β levels in stomach homogenates. **c** *H. pylori* bacterial loads. **d** Stomach weights, **e** gross pathology (arrows showing increased mucosal thickness), **f** corpus mucosal thickness and **g** inflammation scores. **h** PAS-AB-stained sections of gastric tissues from BM-reconstituted mice, with arrows indicating acid mucins (in blue). Representative images from two independent experiments. Scale bars, 1 cm (**e**), 50 µm (**h**). **a–d**, **f**, **g** Each data point represents either an individual mouse (**a–d, g**) or cumulative scores from 2 to 4 sections per stomach section of an individual mouse (**f**) and was combined from two independent experiments. Data correspond to the mean ± SEM. Significance was determined by one-way ANOVA. NS not significant (*p* > 0.05).

To further confirm the epithelial cell origin of IL-18 and assess its role in *H. pylori* infection, we employed a bone marrow (BM) reconstitution mouse model. Consistent with the idea that epithelial cells are a major source of gastric IL-18, γ-irradiated *Il18*^−/−^ recipient mice that were reconstituted with BM cells either from *Il18*^+/+^ or *Il18*^−/−^ donor mice and infected for 5 weeks with *H. pylori* SS1 produced significantly lower levels of IL-18 than *Il18*^+/+^ recipient mice (Fig. 2a; *p* = 0.006 and 0.004, respectively). Similar findings were observed at 18 weeks p.i. (Supplementary Fig. 1a–d). Gastric IL-18 levels were higher in *Il18*^+/+^ mice reconstituted with *Il18*^+/+^ BM but not significantly different from those in *Il18*^+/+^ animals receiving *Il18*^−/−^ BM (Fig. 2a). This finding suggests that haematopoietic cells may produce IL-18 in response to *H. pylori* infection, which is consistent with a previous report showing IL-18 production in lamina propria mononuclear cells isolated from *H. pylori*-infected individuals^6^. Comparable levels of IL-1β and bacterial loads were observed in both *Il18*^+/+^ and *Il18*^−*/*−^ recipient mice, suggesting that epithelial cell-derived IL-18 does not affect IL-1β production nor bacterial colonisation (Fig. 2b, c). Other studies also reported that IL-18 was not required for protection against *H. pylori* infection in mouse challenge^20^ and prophylactic immunisation models^7,20^. Importantly, we observed that mice specifically lacking IL-18 in the non-haematopoietic compartment and reconstituted with BM from *Il18*^+/+^ mice had enlarged stomachs (Fig. 2d, e; *p* = 0.002) with the increased mucosal thickness (gastric hyperplasia; Fig. 2d, e), but not more inflammation (Fig. 2g) when compared with wild-type (WT) animals. The *Il18*^*+/+*^ BM → *Il18*^−/−^ mice also had more acid mucin production than WT animals (Fig. 2h) and displayed increased stomach weights (Fig. 2d; *p* = 0.004) and mucosal thickness (Fig. 2f; *p* = 0.0001) when compared with *Il18*^−*/*−^ BM → *Il18*^−*/*−^ recipient mice. This finding suggests that the production of IL-18 by haematopoietic or radioresistant cells within the BM may be pathogenic in mice unable to produce this cytokine, while these deleterious effects are attenuated by the IL-18 originating from non-haematopoietic cells in *Il18*^*+/+*^ animals.

Similar to the *Il18*^−*/*−^ recipient mice reconstituted with *Il18*^−*/*−^ BM (Fig. 2d), *Il18*^−/−^ total knockout (KO) mice exhibited significantly increased stomach weights at 8 weeks p.i. with *H. pylori* SS1 as compared with *Il18*^+/+^ animals (Supplementary Fig. 1e, f). Despite this difference, however, similar expression levels of the inflammatory cytokine genes, *Il1b* and *Cxcl2* were observed in the *Il18*^+/+^ and *Il18*^−*/*−^ mice (Supplementary Fig. 1g). Consistent with these data, *Il18*^−/−^ mice that were infected for 52 weeks with *Helicobacter felis*, which is more pathogenic than *H. pylori* in mice^21^, also displayed larger stomachs than WT animals (Supplementary Fig. 1h, i). Taken together, our data show that non-haematopoietic cells in the stomach produce IL-18, which plays a protective role against the development of the pre-neoplastic lesions typically observed in chronic *Helicobacter* infection.

### IL-18 responses to *H. pylori* infection are independent of the canonical inflammasome

**Fig. 3:**
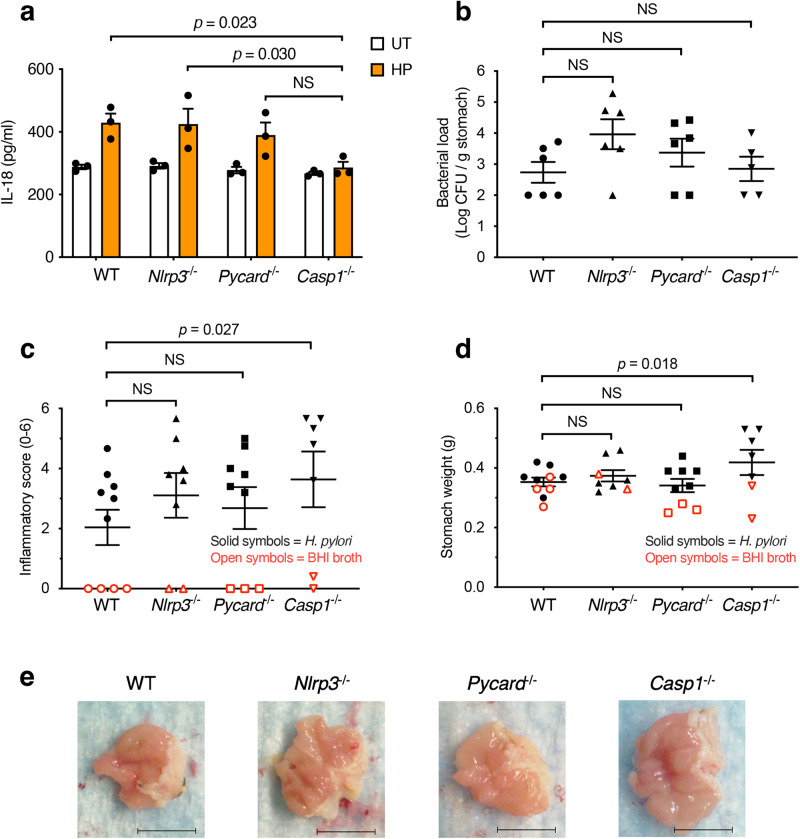
*H. pylori* induces IL-18 production independently of the canonical inflammasome. **a** IL-18 production was assessed in primary gastric epithelial cells that had been isolated from WT, *Nlrp3*^−*/*−^, *Pycard*^−*/*−^ and *Casp1*^−*/*−^ mice and stimulated with *H. pylori* (HP) SS1 bacteria or left untreated (UT). IL-18 levels were determined after 24 h incubation. **b–d** WT and KO mouse strains were infected with *H. pylori* (solid symbols), euthanised at 24 weeks p.i. and then their stomachs were analysed for bacterial loads (**b**), inflammatory scores (**c**), weights (**d**) and gross pathology (**e**). Open symbols in panels **c** and **d** correspond to control mice administered BHI broth alone. Mouse numbers and sexes were as follows: WT (4 males, 6 females), *Nlrp3*^−*/*−^ (8 males), *Pycard*^−*/*−^ (3 males, 6 females) and *Casp1*^−*/*−^ (5 males, 2 females). Scale bar, 1 cm (**e**). Data correspond to the mean ± SEM, with each data point representing a biological replicate (**a**) or individual mouse (**b–d**). Significance was determined by one- or two-way ANOVA (**b–d** and **a**, respectively). NS not significant (*p* > 0.05).

We next sought to determine the inflammasome signalling molecules required for epithelial cell production of IL-18 in response to *H. pylori* infection. *H. pylori* bacteria were added to cells at a multiplicity of infection (MOI) of 10:1 for 1 hr, then washed off and the cells were re-incubated for 23 h. For this, primary gastric epithelial cells were isolated from mice deficient in the canonical inflammasome molecules, Nlrp3, Asc (encoded by the *Pycard* gene) and caspase-1. Similar to primary human gastric epithelial cells^6^, some IL-18 was produced constitutively by primary mouse gastric epithelial cells (Fig. 3a). This may be attributed to the presence of NOD1 ligand (peptidoglycan) in the serum used for tissue culture, which is able to promote low-level NOD1 activation^22^. Primary epithelial cells from *Casp1*^−/−^ mice, but not those from *Nlrp3*^−*/*−^ nor *Pycard*^−*/*−^ animals, produced significantly less IL-18 in response to stimulation with *H. pylori* SS1 bacteria when compared with WT cells (Fig. 3a; *p* = 0.0004). *H. pylori* SS1 bacteria did not induce any IL-18 production in BM-derived macrophages (BMDMs) (Supplementary Fig. 2). As reported previously^23^, the bacterium induced IL-1β responses in BMDMs (Supplementary Fig. 2). These findings underscore the differences in epithelial and myeloid cell responses to *H. pylori* infection.

Next, we performed *H. pylori* SS1 infection studies in WT, *Nlrp3*^−*/*−^, *Pycard*^−*/*−^, and *Casp1*^−/−^ mice. Although all animal groups showed similar levels of bacterial loads at 24 weeks post-infection (Fig. 3b), only *Casp1*^−/−^ mice had increased inflammatory scores (Fig. 3c; *p* = 0.027) and stomach weights (Fig. 3d, e; *p* = 0.018) when compared with WT animals. Moreover, we administered *H. pylori* B128 7.13 bacteria to mice deficient in other known canonical inflammasome molecules, Nlrp1 and Nlrc4, the latter responding to bacterial flagellin and rod proteins^2^, but observed no significant differences in colonisation levels nor stomach pathology when compared with WT animals (Supplementary Fig. 3). Taken together, the findings suggest that the regulation and functions of IL-18 in gastric epithelial cells in response to *H. pylori* infection are dependent on caspase-1, but independent of canonical inflammasome pathways, including the key inflammasome molecules NLRP3 and ASC, which have been implicated in responses to bacterial pathogens.

### NOD1 in epithelial cells mediates IL-18 processing in response to *H. pylori* infection

To further characterise the inflammasome pathway involved in *H. pylori*-induced IL-18 processing, we used human AGS gastric epithelial cells, a standard cell line used in the *H. pylori* field and in which *IL18* mRNA and protein are upregulated in response to *H. pylori* stimulation^6,24^. Consistent with the in vivo data (Fig. 3), we were unable to detect the presence in AGS cells of either NLRP3 or ASC, nor other important inflammasome proteins, NLRC4 or AIM2, either constitutively or in response to *H. pylori* 251 bacteria (Supplementary Fig. 4a–d). These findings suggested that a non-classical inflammasome protein may be involved in *H. pylori*-induced IL-18 processing in human gastric epithelial cells.

**Fig. 4:**
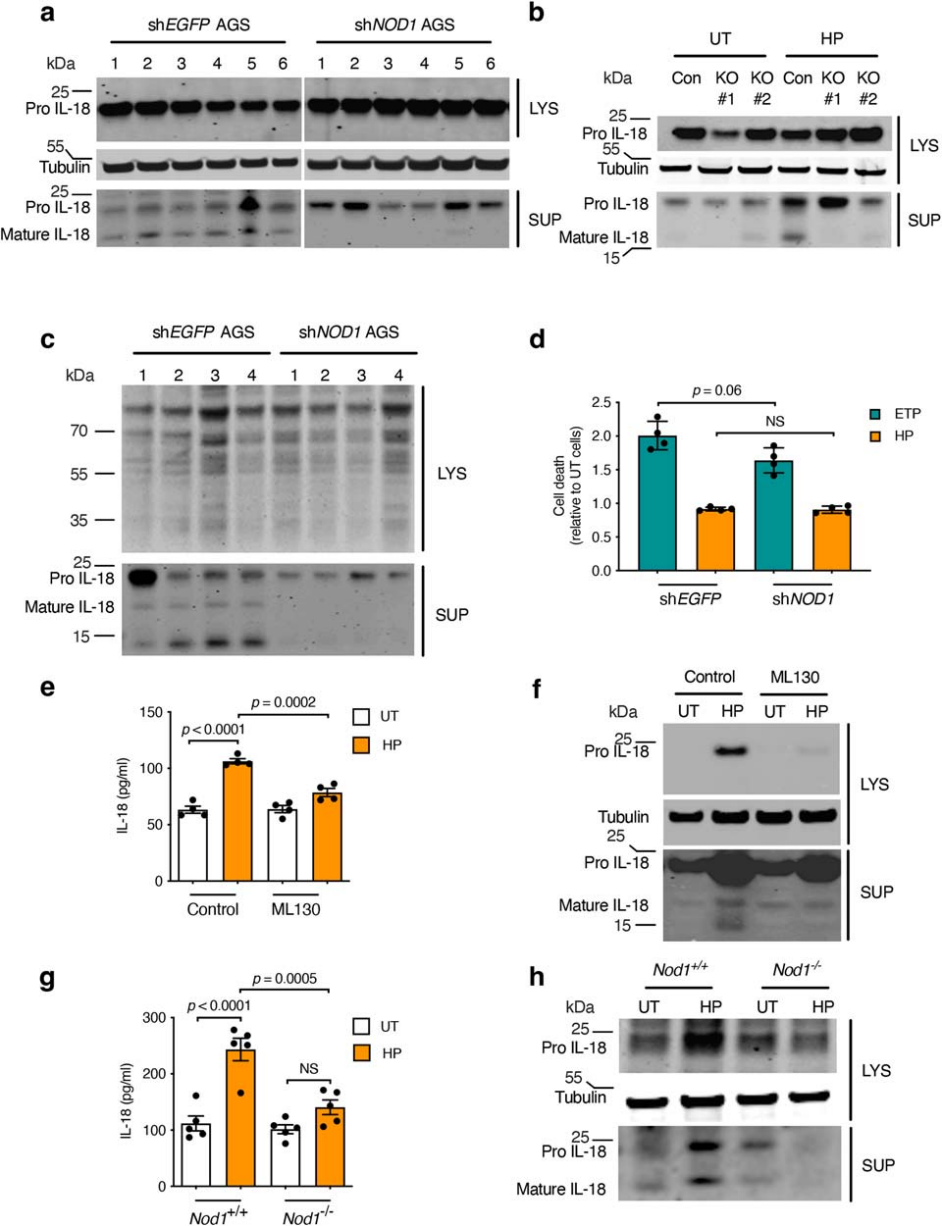
NOD1 is required for *H. pylori*-induced IL-18 processing in epithelial cells. **a–c** Pro- and mature IL-18 production in human AGS gastric epithelial cells. **a** AGS cells stably expressing shRNA to either *EGFP* (sh*EGFP*) or *NOD1* (sh*NOD1*) were left untreated (**1**), or stimulated with *H. pylori* 251WT (**2**), Δ*cag*PAI (**3**) and *cagM* (**4**) mutants, as well as WT 10700 (**5**) or SS1 (**6**) bacteria. **b** AGS *NOD1* CRISPR/Cas9 KO (two clones) or Cas9 control (CON) cells were stimulated with *H. pylori* 251 (HP) or left untreated (UT). **c** sh*EGFP* or sh*NOD1* AGS cells were left untreated (**1**), or stimulated with BHIB medium (**2**), *H. pylori* 10700 (**3**), or SS1 (**4**) MVs (50 µg protein). Tubulin (**a**, **b**) or total protein (**c**) were used as loading controls. **d** Cell death was assessed in sh*EGFP* or sh*NOD1* AGS cells, treated with *H. pylori* 251 (HP) or etoposide (ETP), and assessed relative to that in the respective untreated cells. **e** Mouse GSM06 gastric epithelial cells that had been pre-treated with NOD1 inhibitor (ML130; 5 µM) or vehicle (control) were stimulated with *H. pylori* (HP) SS1 or left untreated (UT). IL-18 levels were measured in culture supernatants. **f** Pro-IL-18 and mature IL-18 were detected in cell lysates (LYS) and supernatants (SUP), respectively. **g**, **h** Primary gastric epithelial cells from *Nod1*^+/+^ and *Nod1*^−/−^ mice were stimulated with *H. pylori* 251 (HP) or left untreated (UT), then analysed for IL-18 production (**g**), pro-IL-18 and mature IL-18 (**h**). IL-18 processing, production and cell death were determined after overnight incubation. In all experiments, *H. pylori* bacteria were added to cells (MOI = 10:1) for 1 h, washed off, and the cells re-incubated for 23 h. Representative images for three independent experiments (**a–c**, **f**, **h**). Mean ± SEM for four (**d**, **e**) or two (**g**) independent experiments. Data in (**g**) are pooled from five primary cell preparations per genotype. Significance was determined by two-way ANOVA (**d**, **e**, **g**). For IL-18 production: in *H. pylori*-stimulated vs. UT control cells (*p* < 0.0001) and *H. pylori*-stimulated cells pre-treated or not with ML130 (*p* < 0.0002; **e**); and *H. pylori*-stimulated vs. UT *Nod1*^+/+^ cells (*p* < 0.0001) and *H. pylori*-stimulated *Nod1*^+/+^ vs *Nod1*^−/−^ cells (*p* < 0.0005; **g**). NS not significant (*p* > 0.05).

We hypothesised that this protein may be NOD1, which plays important roles in the sensing of *H. pylori* bacteria^13,14,17^ and is strongly expressed in AGS cells (Supplementary Fig. 4d). Indeed, interfering with NOD1 signalling in AGS cells, by either short hairpin RNA (shRNA) knockdown (sh*NOD1*^14,25^) or CRISPR/Cas9 knockout (*NOD1* KO)^17^, resulted in the loss of IL-18 processing in response to live *H. pylori* bacteria (Fig. 4a, b). A similar NOD1-dependent effect was observed for IL-18 processing in response to *H. pylori* MVs, which contain peptidoglycan and enter cells to activate NOD1 signalling^14,15^ (Fig. 4c). Some IL-18 processing was also observed in response to the BHI broth (BHIB) medium from which the MVs were isolated. This can most likely be attributed to the serum in the BHIB medium that contains trace amounts of peptidoglycan capable of activating NOD1 signalling^22^. qPCR analyses suggest that the AGS cell line does not produce IL-1β in response to *H. pylori* stimulation (Supplementary Fig. 4e), which is consistent with findings for another human gastric epithelial cell line (MKN7)^26^.

We next studied the contribution of the *H. pylori* T4SS on IL-18 processing using strains lacking a functional T4SS *i.e*. Δ*cag*PAI, Δ*cagM* isogenic mutants and SS1^13^. The levels of mature IL-18 production in cell culture supernatants of control (sh*EGFP*) AGS cells were reduced in response to strains lacking a functional T4SS when compared with the parental clinical isolates, 251 and 10700 (Fig. 4a). The reduced levels of pro-IL-18 production induced in sh*NOD1* AGS cells by the T4SS-deficient strains, when compared with the WT isolates, may be attributed to the ability of the latter to activate T4SS-dependent but NOD1-independent pathways that converge on NF-κB^12^, resulting in the upregulation of pro-IL-18 production. These data show that *H. pylori* T4SS activation of the NOD1 signalling pathway is required for maximal IL-18 production and processing in human gastric epithelial cells. This result is consistent with previous findings for NOD1-dependent IL-8 production^13,14^ and interleukin-33 (IL-33) processing^15^ in human gastric epithelial cells. Importantly, no differences in cell death were observed between the *NOD1* KD and WT AGS cells in response to *H. pylori* stimulation (Fig. 4d), suggesting that the presence of mature IL-18 in culture supernatants was not due to cell lysis.

To further investigate the role of NOD1 in IL-18 processing to *H. pylori* infection, we next used immortalised and primary mouse gastric epithelial cells. Total levels of IL-18 production were increased in the culture supernatants of the mouse GSM06 cell line^27^ in response to *H. pylori* 251 stimulation, whereas it was significantly reduced when NOD1 self-oligomerisation was inhibited by pre-treatment with the NOD1-specific inhibitor, ML130^28^ (Fig. 4e; *p* = 0.0002). Pre-treatment of cells with ML130 also blocked the formation of both precursor and mature forms of IL-18 in response to *H. pylori* SS1 stimulation (Fig. 4f). Similarly, primary gastric epithelial cells from *Nod1*^−*/*−^ mice stimulated with *H. pylori* SS1 bacteria produced significantly lower levels of total IL-18 (Fig. 4g; *p* = 0.0005), as well as pro- and mature IL-18 (Fig. 4h), when compared with *Nod1*^+/+^ cells. As shown by the responses to *H. pylori* SS1 bacteria, a functional T4SS was not required for the induction of IL-18 processing in murine epithelial cells (Fig. 4f, h). This finding is consistent with our previous work in which *H. pylori* SS1 bacteria mediated NOD1-dependent regulation of IL-33 responses in murine primary gastric epithelial cells^17^. We also found that Nod1 was not required for IL-18 (nor IL-1β) production by BMDMs in response to either *H. pylori* SS1 bacteria or the canonical inflammasome activators, LPS and nigericin (Supplementary Fig. 5), again indicating that NOD1 mediates IL-18 processing in a cell-specific manner.

### NOD1 mediates IL-18 processing independently of RIPK2–NF-κB signalling

**Fig. 5:**
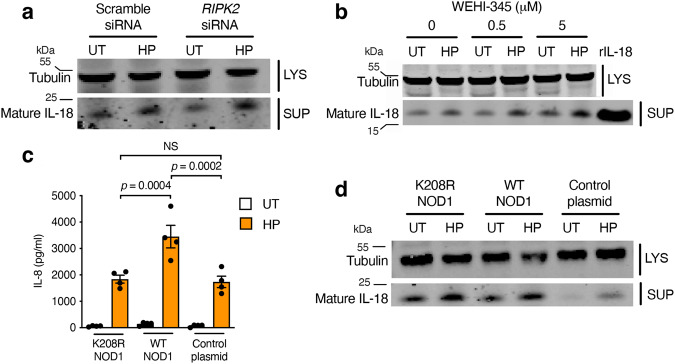
The NOD1 adaptor protein RIPK2 is dispensable for NOD1-dependent IL-18 processing in epithelial cells. **a** Human AGS gastric epithelial cells were either pre-treated with scramble or RIPK2 siRNA and then stimulated with *H. pylori* (HP) 251 or left untreated (UT). IL-18 processing was assessed in culture supernatants (SUP). LYS = cell lysates. **b** AGS cells were pre-treated with varying concentrations of the RIPK2 inhibitor WEHI-345 and then stimulated or not with *H. pylori*. **c**, **d** shNOD1 AGS cells were transfected with either a mutant form of NOD1 (K208R NOD1), WT NOD1 or the vector (control plasmid). IL-8 production (**c**) and IL-18 processing (**d**) were assessed in culture supernatants after 24 h incubation. Representative images for three independent experiments (**a**, **b**, **d**). Mean ± SEM for four independent experiments (**c**). Significance was determined by one-way ANOVA. NS not significant (*p* > 0.05).

Classically, NOD1 activation results in the recruitment of the adaptor molecule RIPK2, induction of the NF-κB signalling pathway and upregulation of pro-inflammatory cytokine production^13,14,16,17^. We investigated the role of RIPK2 in *H. pylori*-induced IL-18 processing in epithelial cells by small interfering RNA (siRNA) transfection or blocking its activity with the kinase inhibitor, WEHI-345^29^. siRNA-mediated KD of *RIPK2* was confirmed by qPCR detection of *RIPK2* expression and by measuring IL-8 production as a read-out for classical NOD1 signalling^17^. Blocking RIPK2 signalling had no effect on IL-18 processing in response to *H. pylori* 251 stimulation in AGS cells (Fig. 5a, b). In agreement with this finding, IL-18 processing was unaffected in both primary gastric epithelial cells and BMDMs from *Ripk2*^−/−^ mice, whereas epithelial cell production of the NOD1-regulated chemokines, Cxcl1/keratinocyte chemoattractant (KC) and Cxcl2/macrophage inflammatory protein 2 (MIP2)^13,15,30,31^, was reduced in response to *H. pylori* 251 stimulation (Supplementary Fig. 6; *p* = 0.0005 and *p* = 0.0002, respectively).

To demonstrate that NF-κB activation by NOD1 is dispensable for IL-18 processing in epithelial cells, we restored NOD1 expression in sh*NOD1* AGS cells with plasmid constructs expressing either WT or a mutant form of NOD1 (K208R NOD1) that is unable to activate NF-κB signalling^32^. As expected, NOD1 sh*NOD1* AGS cells transfected with plasmid encoding WT NOD1 exhibited significantly higher IL-8 responses to *H. pylori* 251 stimulation than cells transfected with either the K208R NOD1 mutant NOD1 or empty control plasmids (Fig. 5c; *p* = 0.0004 and *p* = 0.0002, respectively). Conversely, WT and K208R mutant NOD1 plasmids were equally able to rescue IL-18 processing in sh*NOD1* AGS cells (Fig. 5d), suggesting that NOD1 mediates gastric epithelial IL-18 processing in an NF-κB-independent manner. We propose that *H. pylori* induce IL-18 processing via a non-classical type of NOD1 signalling pathway.

It was recently reported that NOD1 plays a non-redundant role with the host adaptor protein, TIFA, in regulating inflammatory responses by gastric epithelial cells to *H. pylori*^33^. Consistent with this finding, we showed that transfection with *TIFA* siRNA reduced IL-8 responses to *H. pylori* 251 bacteria in AGS cells when compared with scramble siRNA (Supplementary Fig. 7a, b). Moreover, *TIFA* siRNA transfection abrogated IL-18 processing in AGS cells stimulated with *H. pylori* bacteria (Supplementary Fig. 7c), thus suggesting that cross-talk between NOD1 and TIFA pathways may be important for *H. pylori*-induced IL-18 processing in epithelial cells.

### NOD1 activates caspase-1 to promote IL-18 processing in epithelial cells

It is well established that caspase-1 is a key protease responsible for the cleavage of pro-IL-18 to its mature form in innate immune cells^1,19^. Although early studies reported potential CARD–CARD interactions between NOD1 and pro-caspase-1^32^, these findings have never been re-visited.

**Fig. 6:**
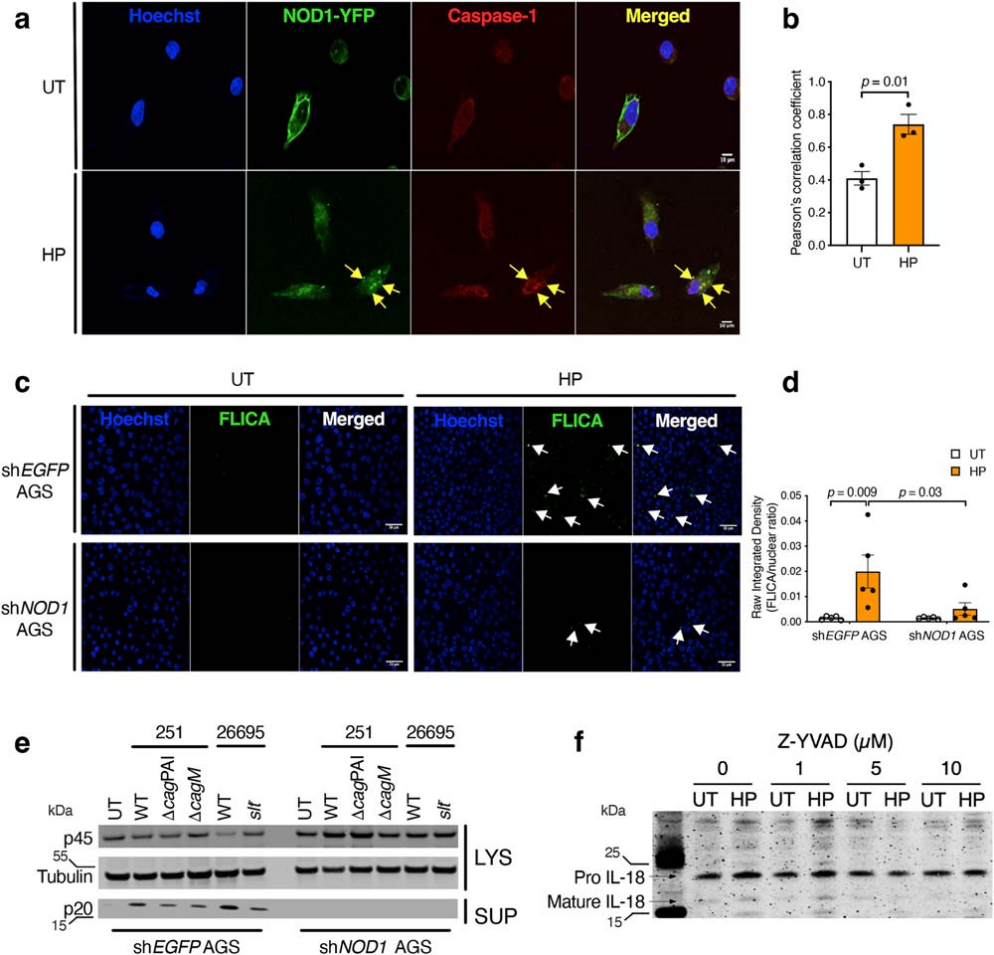
NOD1 activates caspase-1 to promote IL-18 processing in epithelial cells. **a** Human AGS gastric epithelial cells stably expressing shRNA to *NOD1* (sh*NOD1*) were transfected with YFP-labelled NOD1, stimulated with *H. pylori* (HP) 251 or left untreated (UT), then incubated with an anti-caspase-1 antibody. In response to *H. pylori* stimulation, NOD1 and caspase-1 molecules can be observed co-localising (arrows). Cell nuclei were stained with Hoechst 33342, the images merged and the sections were analysed by confocal microscopy. Scale = 10 µm. **b** Co-localisation of NOD1-YFP and caspase-1 was quantified and analysed using Pearson’s correlation coefficient. **c** AGS cells stably expressing shRNA to either *EGFP* (sh*EGFP*) or *NOD1* (sh*NOD1*) were stimulated with *H. pylori* or left untreated and then incubated with FLICA, a fluorescently labelled caspase inhibitor that binds irreversibly to caspase-1. Increased levels of fluorescence can be seen in the sh*EGFP* cells responding to *H. pylori* stimulation (arrows). Cell nuclei were stained with Hoechst 33342 and the images merged. Scale bar = 50 µm. **d** FLICA fluorescence is expressed as the raw integrated density values, determined as a ratio of the total numbers of cell nuclei, using Fiji software. **e** sh*EGFP* and sh*NOD1* AGS cells were left untreated (UT), or stimulated with the indicated *H. pylori* WT and mutant strains (Δ*cag*PAI, *cagM*, *slt*). Pro- (p45) and mature (p20) forms of caspase-1 were detected by Western blotting of cell lysates (LYS) and supernatants (SUP). **f** AGS cells were pre-treated with varying concentrations (0–10 µM) of the caspase-1 inhibitor Z-YVAD, then left untreated (UT) or stimulated with *H. pylori* (HP). IL-18 processing was determined by Western blotting. All assays were performed after 24 h incubation. Two independent experiments. Representative images (**a**, **c**, **e**, **f**). Mean ± SEM for 3 (**b**) or 5 (**d**) fields analysed. Significance was determined by a two-tailed unpaired *T*-test (**b**) or two-way ANOVA (**d**).

Therefore, to examine whether NOD1 mediates IL-18 processing in epithelial cells via caspase-1 activation, sh*NOD1* AGS cells that were transiently expressing yellow fluorescent protein (YFP)-labelled NOD1 were incubated with anti-caspase-1 antibody. As can be observed (Fig. 6a), NOD1 is normally proximal to the cell membrane under basal conditions, but moved towards the cytosol and formed yellow punctate stained aggregates with endogenous caspase-1, in response to *H. pylori* 251 stimulation (Fig. 6a). Quantitative analyses of the images revealed significant levels of co-localisation between NOD1-YFP and caspase-1 in *H. pylori*-stimulated cells (Fig. 6b; *p* = 0.01). This suggested that NOD1 may associate with caspase-1 upon activation by *H. pylori*. To demonstrate NOD1-mediated activation of caspase-1, we used the fluorescently labelled inhibitor of caspase (FLICA) reagent that binds irreversibly to the active form of caspase-1. WT (sh*EGFP*) AGS cells exhibited enhanced staining with FLICA after stimulation with *H. pylori* 251, whereas staining was barely detected in sh*NOD1* AGS cells (Fig. 6c). Significant increases in FLICA staining were observed between *H. pylori*-stimulated sh*EGFP* AGS cells when compared with both untreated shEGFP cells (Fig. 6d; *p* = 0.009) and *H. pylori*-stimulated sh*NOD1* cells (Fig. 6d; *p* = 0.03), confirming that caspase-1 activation is *H. pylori*-/NOD1-dependent. Moreover, we could only detect the mature, active form of caspase-1 (p20) in the culture supernatants of WT but not sh*NOD1* AGS cells in response to stimulation with the T4SS-functional *H. pylori* 251 and 26695 strains (Fig. 6e). We also observed reduced levels of active caspase-1 in AGS cells stimulated with *H. pylori* mutant strains lacking either a functional T4SS (Δ*cag*PAI or Δ*cag*M) or defective in activating the NOD1 pathway (*slt*^−^)^13^ (Fig. 6e). Inactivation of caspase-1 with the inhibitor Z-YVAD-fmk resulted in a dose-dependent reduction of mature IL-18 production in cells (Fig. 6f). The data suggest that *H. pylori* activation of the NOD1 pathway contributes to caspase-1 activation in gastric epithelial cells, but that other pathway(s) may also be involved. Collectively, these data show that NOD1 sensing of *H. pylori* promotes IL-18 processing via activation of caspase-1.

### NOD1 interacts with caspase-1 to promote maximal IL-18 processing

**Fig. 7:**
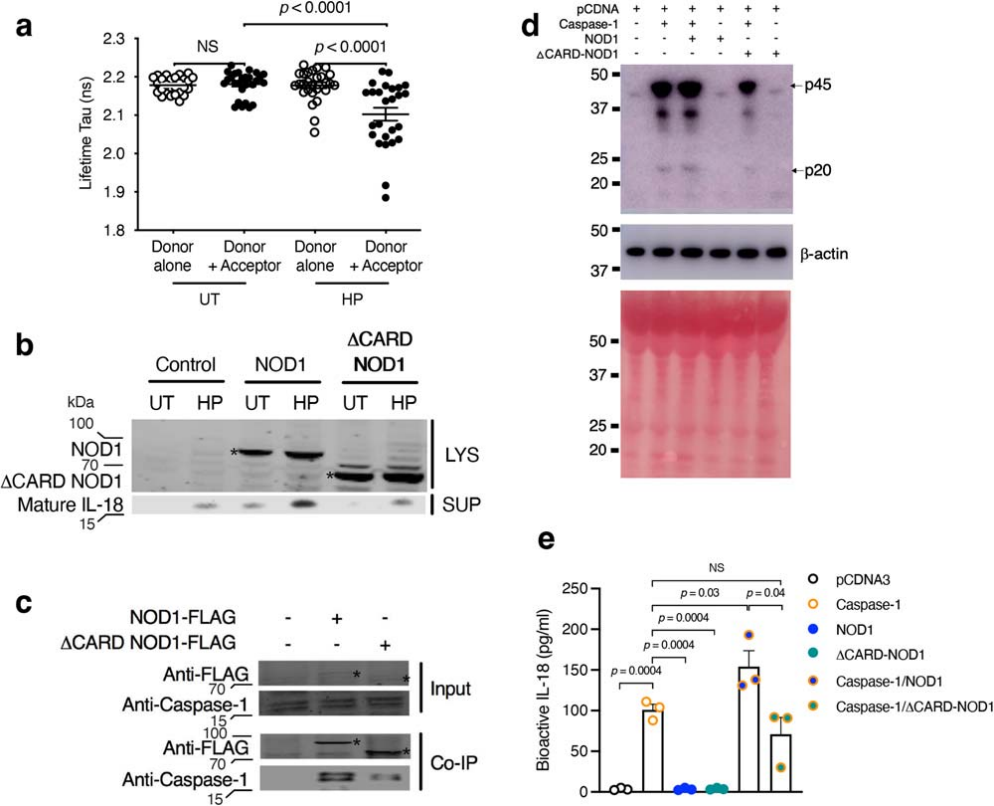
NOD1 interacts with caspase-1 to promote bioactive IL-18 production. **a** Human AGS gastric epithelial cells stably expressing shRNA to *NOD1* (sh*NOD1*) were transfected with NOD1-mOrange plasmid (Donor alone) or both NOD1-mOrange and caspase-1-GFP (Donor + Acceptor). Cells were then stimulated with *H. pylori* (HP) 251 or left untreated (UT) and NOD1**-**caspase-1 interactions determined by the FLIM-FRET technique. A decrease in the lifetime of the donor fluorochrome, indicative of quenching of the donor by the acceptor, indicates close proximity (<10 nm) between NOD1 and caspase-1 proteins. **b–e** *NOD1* KO AGS cells, generated by CRISPR/Cas9 gene editing, were transfected with either full-length NOD1 (NOD1), CARD-deficient NOD1 (ΔCARD-NOD1), or caspase-1, then stimulated with *H. pylori* or left untreated. As a negative control, cells were transfected with pCDNA3 (**d**). **b** NOD1 and mature IL-18 were detected in cell lysate (LYS) or supernatant (SUP) samples of the transfected cells. **c** Lysates of transfected cells (input) were probed with antibodies to detect either FLAG-tagged proteins or endogenous caspase-1, then subjected to immunoprecipitation using an anti-FLAG antibody. The immunoprecipitated proteins were probed with anti-FLAG or anti-caspase-1 antibodies (Co-IP). **d**, **e** Transfected cells were analysed for caspase-1 processing (**d**) and secreted (bioactive) IL-18 production (**e**). **d** Pro (p45) and mature (p20) forms of caspase-1 were detected by Western blotting. Protein loadings were confirmed by β-actin detection and Ponceau S staining. **e** Levels of bioactive IL-18 production were determined using transfected cell supernatants analysed in an IL-18 reporter cell line. All assays were performed after 24 h incubation. Ten random fields of view were recorded per treatment with a minimum of five cells per field analysed (**a**). Representative data for two independent experiments (**b–d**). Mean ± SEM of duplicate values for three independent experiments (**e**). Significance was determined by one-way ANOVA (**a**) or two-sided Student’s *t*-test (**e**). UT vs. *H. pylori*-simulated cells with donor and acceptor (*p* < 0.0001*)*; *H. pylori*-simulated cells with donor alone vs. donor and acceptor (*p* < 0.0001) (**a**). NS = not significant. Cells transfected with: caspase-1 vs. pCDNA3, NOD1 or ΔCARD-NOD1 (all *p* = 0.0004); caspase-1 vs. caspase-1/NOD1 (*p* = 0.03), caspase-1/ΔCARD-NOD1 (NS); and caspase-1/NOD1 vs. caspase-1/ΔCARD-NOD1 (*p* = 0.04) (**e**).

We next sought to elucidate potential NOD1-caspase-1 molecular interactions and their involvement in IL-18 processing. It is well established that NLRP3 and AIM2 need the adaptor protein, ASC, to recruit and activate caspase-1. However, as reported above (Supplementary Fig. 4), the AGS cell line does not produce detectable levels of ASC protein, suggesting that NOD1 does not require ASC to mediate IL-18 processing in epithelial cells. Thus, we hypothesised that NOD1 may instead interact with caspase-1, possibly via homotypic CARD–CARD interactions. This hypothesis was addressed using the fluorescence lifetime imaging microscopy-Förster resonance energy transfer (FLIM-FRET) technique in live cells. For this, we transfected sh*NOD1* AGS cells with NOD1-mOrange plasmid (donor alone control) or both NOD1-mOrange and the acceptor plasmid, encoding caspase-1-GFP (Fig. 7a). We found that *H. pylori* 251 stimulation led to a significant reduction in the lifetime of the donor fluorochrome, indicative of quenching of the donor by the acceptor caspase-1-GFP due to close proximity (<10 nm) of NOD1 and caspase-1 proteins (Fig. 7a; *p* < 0.0001). In contrast, the lifetime of the donor remained unchanged in unstimulated cells. Reconstitution of NOD1 expression with full-length NOD1 in *NOD1* KO AGS cells resulted in maximal IL-18 processing in response to *H. pylori* 251 bacteria (Fig. 7b). In contrast, cells transfected with ΔCARD-NOD1 exhibited background levels of IL-18 processing (Fig. 7b), which was most likely due to the effect of *H. pylori* on pro-IL-18 synthesis. To further investigate NOD1–caspase-1 interactions, we performed immunoprecipitation experiments in which *NOD1* KO AGS cells were transfected with either NOD1-FLAG or ΔCARD-NOD1-FLAG. As shown in Fig. 7c, endogenous caspase-1 could be immunoprecipitated in cells transfected with NOD1-FLAG and, to a lesser extent, in those transfected with the ΔCARD form of the protein.

Finally, to determine whether overexpression of NOD1 and caspase-1 would be sufficient to promote IL-18 processing, *NOD1* KO AGS cells were transfected with constructs expressing either caspase-1, NOD1-FLAG, ΔCARD-NOD1-FLAG, or combinations of these constructs (Fig. 7d, e). Co-expression of NOD1 with caspase-1 resulted in the production of bioactive (secreted) IL-18, as measured using an IL-18 reporter system in HEK293 cells. Importantly, the levels of bioactive IL-18 induced by NOD1/caspase-1 co-expression were significantly higher than those induced by either caspase-1 expression alone (*p* = 0.03) or co-expression of caspase-1 and ΔCARD-NOD1 (*p* = 0.04) (Fig. 7e), suggesting that the CARD of NOD1 is required for *maximal* IL-18 processing. Collectively, these results suggest that NOD1 interacts with caspase-1 to promote IL-18 processing in epithelial cells responding to *H. pylori* infection and that these interactions occur, at least in part, via CARD–CARD interactions.

### NOD1 maintains epithelial homoeostasis in response to *H. pylori* infection

**Fig. 8:**
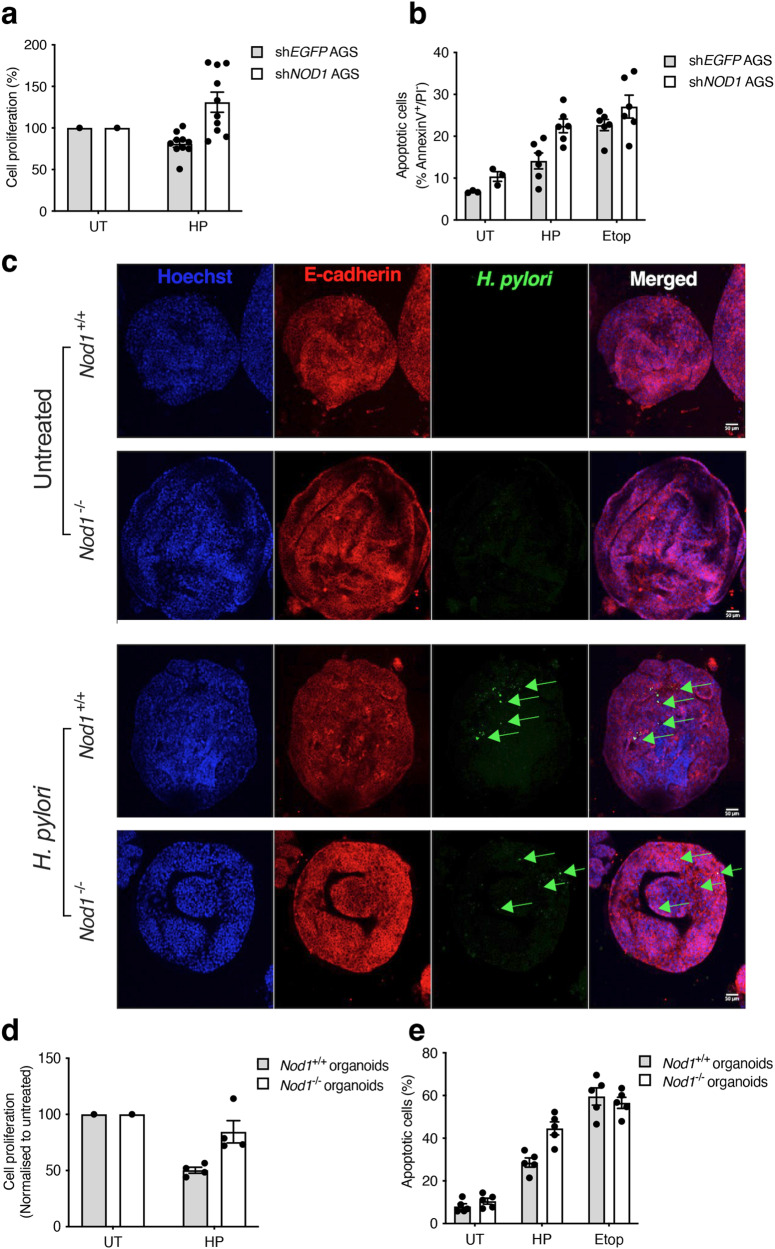
NOD1 maintains epithelial homoeostasis in response to *H. pylori* infection. **a**, **b** Human AGS gastric epithelial cells stably expressing shRNA to either *EGFP* (sh*EGFP*) or *NOD1* (sh*NOD1*) were stimulated with *H. pylori* (HP) 251 or left untreated (UT). Cell proliferation (**a**) and apoptosis (**b**) were determined using the MTT assay and Annexin V/PI staining, respectively. As a control, cells were treated with the apoptotic-inducing agent, etoposide (Etop; 5 μM). **c–e** Gastric organoids were isolated from *Nod1*^*+/+*^ and *Nod1*^−*/*−^ mice, then either left untreated (UT) or treated with *H. pylori* (HP) or etoposide (Etop). *H. pylori* bacteria (green) were microinjected into the lumen of organoids. **c** Confocal microscopy images of *Nod1*^*+/+*^ and *Nod1*^−*/*−^ organoids showing E-cadherin^+^ epithelial cells (red) with nuclei (blue) stained by Hoechst 33342. Merged images show *H. pylori* bacteria (green; arrows) within the lumen of organoids. **d**, **e** Gastric organoid cultures from *Nod1*^*+/+*^ and *Nod1*^−*/*−^ mice were assessed for changes in cell proliferation (**d**) and apoptosis (**e**) in response to *H. pylori* or etoposide treatment, using Presto Blue assay and Annexin V/propidium iodide staining, respectively. All assays were performed after 24 h incubation. Data correspond to the mean ± SEM. Two independent experiments. Representative images (**c**) and mean ± SEM for combined data in which each point corresponds to a replicate (**a**, **b**, **d**, **e**).

As IL-18 appeared to be important for protecting tissues against excessive pathology during chronic *H. pylori* infection in vivo (Fig. 2, Supplementary Fig. 1), we next asked whether NOD1-mediated IL-18 secretion may play a role in regulating gastric epithelial cell survival responses. For this, we performed in vitro assays including MTT assay and Annexin V/propidium iodide (PI) staining to assess cell proliferation and apoptosis, respectively. sh*NOD1* KD AGS cells exhibited higher levels of both cell growth and apoptosis in response to *H. pylori* stimulation when compared with sh*EGFP* AGS cells (Fig. 8a, b). Differences in apoptosis were not observed in cells stimulated with the apoptotic-inducing agent, etoposide (Fig. 8b). These data suggest that NOD1 is involved in regulating both epithelial cell growth and death in response to *H. pylori* stimulation. To confirm these findings, we established a 3-D gastric organoid model from *Nod1*^*+/+*^ and *Nod1*^−*/*−^ mice. *H. pylori* 251 bacteria were added to the lumen of the organoids by microinjection (Fig. 8c). Consistent with data for AGS cells (Fig. 8a, b), *Nod1*^−/−^ organoids exhibit higher levels of cell proliferation and apoptosis, as compared with *Nod1*^+/+^ organoids (Fig. 8d, e). Collectively, these results show that NOD1 is important for maintaining homoeostasis in gastric epithelial cell turnover in response to *H. pylori* infection.

## Discussion

Current understanding of canonical inflammasome functions has largely been shaped by studies in haematopoietic cells and, in particular, the processing of the pro-inflammatory cytokine, IL-1β. In contrast, the functions of inflammasomes in non-haematopoietic cell lineages, including epithelial cells, are much less well understood^3^. The inflammasome molecules AIM2, NLRP3, NLRP6 and NLR Family Apoptosis Inhibitory Protein (NAIP)/NLRC4 were reported to play important roles in epithelial cells by protecting against infection and maintaining tissue homoeostasis^3,34–38^. An important distinction between epithelial inflammasomes and those in mononuclear phagocytes is that the former is primarily involved in IL-18 production, whereas the latter is a major source of IL-1β^3^. IL-18 was initially identified as promoting intestinal inflammation, but there is now overwhelming evidence to support the contention that this cytokine can also play protective roles against colitis^3,36,39–41^. In the current study, we show that IL-18 plays a protective role against the development of pre-neoplastic changes in the stomach due to chronic *H. pylori* infection. Moreover, we identify a new type of inflammasome, involving NOD1, as being important for epithelial cell production of mature, biologically active IL-18 in response to *H. pylori* infection. Interestingly, it was reported that *Chlamydophila trachomatis* also promotes IL-1 family cytokine processing in a NOD1-dependent and NALP3/ASC-independent manner^42^, however, that work studied IL-1β and not IL-18 secretion. Furthermore, IL-1β production was assessed in a specialised cell type (trophoblasts) and not epithelial cells.

Intestinal epithelial cells constitutively produce IL-18, suggesting that epithelial inflammasomes have a “ready-to-go” phenotype that allows rapid responses to pathogens and other damage to the gut^3,36^. In agreement with this suggestion, we (Figs. 4 and 5) and others^4–6^ have established that IL-18 is expressed constitutively in the gastric mucosa, with gene expression and protein levels upregulated in response to *H. pylori* infection. Nevertheless, the role of IL-18 responses in *H. pylori* infection and associated diseases is unclear. A gene polymorphism study identified certain haplotypes as possibly being associated with susceptibility to *H. pylori* infection in the Korean population^43^, but functional data for IL-18 responses were not provided. Other studies could not find any correlations between IL-18 haplotype, phenotype, and clinical outcome, including gastric cancer^4,44^. Conversely, a correlation was found between certain IL-18 haplotypes, higher gastric IL-18 levels and more severe mononuclear cell infiltration in *H. pylori*-infected subjects^4^. Nevertheless, this does not prove causality and may simply reflect IL-18 production by the mononuclear cells recruited to the site of infection. Yamauchi et al. isolated mononuclear and epithelial cells from the gastric mucosa and showed that both cell types produce IL-18 in response to *H. pylori* stimulation ex vivo^6^, but were unable to determine the relative contributions of these cells to IL-18 production in vivo. We found that haematopoietic cells produce low levels of IL-18 (Figs. 1c, d and 2a). Interestingly, however, when IL-18-producing haematopoietic cells were transferred to *Il18*^−/−^ mice, we observed increased stomach weights and mucosal thickness in the animals (Fig. 2d, e), suggesting a potentially pathogenic role for these cells in the context of *H. pylori* infection. Nevertheless, our data provide unequivocal evidence that epithelial cells are an important source of IL-18 production in the stomach during *H. pylori* infection (Figs. 1c, d and 2a, b). Using primary and immortalised cell models, we show that epithelial cells can process IL-18 to its mature form in response to *H. pylori* bacteria or MVs, which contain peptidoglycan (Fig. 4a–c, f, h). We suggest that to better understand the role of IL-18 in *H. pylori* disease, it is necessary to not only investigate the cellular source of this cytokine but also the forms (pre-formed and mature, rather than just the total amount) that are present in the gastric tissues from *H. pylori*-infected subjects presenting with diseases of varying severity.

It has been reported by several groups that *H. pylori* induce NLRP3 inflammasome activation and IL-1β responses in dendritic cells, neutrophils and macrophages/monocytes^23,45–49^. *H. pylori* was shown to upregulate IL-18 production in these immune cells^7,46–48^ via an NLRP3-dependent mechanism^47,48^. Similarly, we observed NLRP3-dependent regulation of IL-1β and IL-18 responses in BMDMs (Supplementary Fig. 2); however, no role could be found for this inflammasome, nor for its adaptor ASC, in IL-18 production by primary mouse gastric epithelial cells (Fig. 3a). *H. pylori* upregulated IL-18 production and processing in the human AGS gastric epithelial line (Figs. 4e, f and 5d) which, contrary to previous reports^50,51^, does not appear to express either NLRP3 or ASC (*PYCARD*) (Supplementary Fig. 4). These findings are confirmed by another study, which also reported that AGS cells do not produce ASC^6^. RNA sequencing data also revealed very low expression levels of *NLRP3* and *PYCARD* in the AGS cell line, equivalent to 0.01 and 0.54 reads/kilobase of transcript/million mapped reads (RPKM), respectively (GEO database Accession number GSM1056529). Consistent with these data, no differences in *H. pylori* bacterial loads or resulting inflammation were observed in *Nlrp3*^−/−^ or *Pycard*^−/−^ mice, when compared with WT animals (Fig. 3b–d). One group reported increased bacterial loads and decreased gastric inflammation in *Nlrp3*^−/−^ mice^23^, whereas the complete opposite was reported in another study^46^. The reasons for the divergent results between these three studies are unclear but may stem from differences in the composition of the mouse microbiota and not from the bacterial strains used, as the latter two studies used the same strain, i.e*. H. pylori* 10700 (PMSS1). Notwithstanding the different findings, the data presented here clearly establish that the canonical NLRP3/ASC inflammasome is not required for gastric epithelial cell production of IL-18 responses to *H. pylori* infection (Figs. 3 and 4e, f).

Additionally, our data suggest that the inflammasome protein NLRC4 does not play a significant role in the infection (Supplementary Fig. 3g, h). This finding is compatible with the known inability of *H. pylori*^23^ or its flagellin^52^ to promote caspase-1 processing and IL-1β secretion in macrophages. Our findings are, however, different to those of another group who recently reported that NLRC4 is important for *H. pylori* T4SS-dependent induction of IL-18 responses in gastric epithelial cells, using primary gastric epithelial cells from WT and *Nlrc4*^−/−^ mice^26^. The authors used the *H. pylori* PMSS1/10700 strain, which, like the B128 7.13 strain used here (Supplementary Fig. 3g, h), has a functional T4SS^53^. It is important to also note that although the authors demonstrated that the MKN7 human gastric epithelial cell line produced IL-18 in response to *H. pylori*, they did not confirm a role for NLRC4 signalling in these cells by siRNA or CRISPR/Cas9 gene editing. Our work (Supplementary Fig. 4) and RNA sequencing data suggest that AGS cells express very low levels (0.58 RPKM), if any, of *NLRC4* (GEO database Accession number GSM1056529). Although it cannot be discounted that NLRC4 plays a role in the regulation of inflammation in vivo via epithelial production of IL-18^26^, further mechanistic insights are required to understand how *H. pylori* might activate NLRC4 signalling in gastric epithelial cells.

A key and novel discovery of the current work is the observation that NOD1 interacts with caspase-1 to mediate *H. pylori* induction of IL-18 processing in epithelial cells. Interestingly, this IL-18 processing occurs independently of interactions between NOD1 and its adaptor molecule, RIPK2 (Fig. 5). Early studies in the field reported NOD1 interactions with caspase-1^32,54^, resulting in IL-1β secretion in cells^54^. A caveat to those studies, however, is that they were undertaken using over-expression approaches that can be susceptible to experimental artefacts. We now provide several lines of supportive evidence indicating that NOD1 interacts with caspase-1 to mediate IL-18 responses in epithelial cells. Firstly, we have confirmed the findings of previous investigations by performing similar over-expression experiments (Fig. 7b). Secondly, we performed immunoprecipitation with anti-FLAG antibodies to pull down caspase-1 associated with NOD1-FLAG and ΔCARD NOD1-FLAG proteins (Fig. 7c) and fluorescence imaging to show close interactions between NOD1 and caspase-1 in response to *H. pylori* stimulation (Figs. 6a–d and 7a). Thirdly, we demonstrate that caspase-1 processing is itself dependent on NOD1 (Fig. 6e) and that co-expression of caspase-1 and NOD1 induced maximal bioactive IL-18 production (Fig. 7e). Fourthly, we show that IL-18 production is abrogated in primary gastric epithelial cells from *Casp1*^−/−^ mice (Fig. 3a) and in cells pre-treated with the caspase-1 inhibitor, Z-YVAD-fmk (Fig. 6f). Finally, *Casp1*^−/−^ mice infected with *H. pylori* reproduce aspects of the severe pathology observed in *Il18*^−/−^ animals (Fig. 3b–e), consistent with the enhanced inflammatory changes also observed in *Casp1*^−/−^ mice infected with *H. felis*^7^.

NLRs were originally identified for their roles in host defence and inflammation. However, more recent work in colitis models has shown that these proteins also play important roles in tissue repair and the maintenance of homoeostasis. NLRs were shown to mediate these protective effects via various mechanisms, including the production of antimicrobial peptides, control of the gut microbiota^35,37^, prevention of tumour development^34,55^ and regulation of NF-κB and MAPK signalling^56^. A key downstream mediator of these tissue-protective responses is IL-18^35,37,57^. Indeed, administration of IL-18 to *Casp1*^−/−^ mice protected animals from tissue damage and death caused by dextran sulfate sodium treatment^57^. We propose that IL-18 may play a similar protective role against tissue damage caused by chronic *H. pylori* infection. Our data show that NOD1 is an important regulator of bioactive IL-18 production by epithelial cells in response to the infection and, moreover, that this NLR plays a protective role in tissue homoeostasis through its control of cell proliferation and apoptosis (Fig. 8). Supportive evidence for this suggestion comes from the Mongolian gerbil model in which treatment with a NOD1 agonist prior to *H. pylori* challenge was shown to suppress the development of inflammation and carcinogenesis in animals^31^. Future studies are warranted to determine whether the tissue-protective role of *H. pylori* infection is dependent on the NOD1–caspase-1–IL-18 signalling axis.

In conclusion, we have identified a new NOD1 inflammasome pathway that mediates different cellular responses to those regulated by classical NOD1–RIPK2–NF-κB signalling. We also identified the existence of potential cross-talk between this NOD1 inflammasome pathway and the cytosolic adaptor protein, TIFA (Supplementary Fig. 7), which was previously reported to play a non-redundant role with NOD1 in mediating inflammatory responses by epithelial cells to *H. pylori* and *Shigella flexneri*^33^. Similar to other NLR inflammasomes, activation of NOD1 signalling via a single stimulus (i.e. *H. pylori*) promotes both inflammatory and tissue repair responses in cells. These responses occur simultaneously in cells in vitro but may be temporally regulated in vivo, particularly over long periods, such as chronic *Helicobacter* infection. This regulation may occur via the actions of CARD-containing proteins that have been shown to differentially regulate inflammatory and cell death pathways^58^. Indeed, it was shown that the CARD-containing protein, CARD6, could selectively modulate NOD1/NF-κB signalling, independently of caspase-1/IL-1β secretion^59^. Interrogation of the STRING database identified two protein interaction nodes for NOD1: one node comprising proteins associated with inflammatory responses (e.g. RIPK2) and autophagy (e.g. ATG16L1); and the other comprising proteins, including caspase-1, involved in cell death pathways (Supplementary Fig. 8). Future studies are required to determine how NOD1 is able to regulate these different downstream signalling pathways, as well as its interactions with these different proteins. We propose that other Gram-negative bacteria that colonise mucosal surfaces and mediate NOD1 signalling (e.g. *Pseudomonas aeruginosa*) may promote tissue repair by similar mechanisms.

## Methods

### Mouse strains, cell lines and bacterial strains

*Casp1*^−*/*−^*(Casp1*^−*/*−^*Casp11*^−*/*−^)^60^, *Il18*^−*/*−^^60^, *Nlrp1*^−*/*−^^61^*, Nlrp3*^−*/*−^^62^, *Nlrc4*^−/−^^63^, *Pycard*^−/−^^60^*, Ripk2*^−/−^^29^ and WT C57BL/6J mice were bred as litter mates at WEHI. *Nod1*^−*/*^^17^, *Nod1*^fl/fl^ and *Nod1*^fl/fl^ × LysM-Cre mice (Supplementary Fig. 9) and WT mice were bred at the Animal Research Facility, Monash Medical Centre (MMC). KO mice had been backcrossed at least ten generations on the C57BL/6J background and maintained under specific pathogen-free conditions in micro-isolator cages with access to food and water ad libitum, under controlled temperature (18–22 °C) and humidity (50–60%) and a 12-h dark/12-h light cycle. Experiments were performed using mice of both sexes and age-matched (3-8 weeks of age). Mouse primary gastric epithelial cells^17^ and BMDMs^60^ were isolated, as described previously. Permission was granted to perform animal experimentation by the Walter and Eliza Hall Institute of Medical Research Animal Ethics Committee (2014.004, 2011.014, 2008.022) and Animal Ethics Committee A at Monash Medical Centre (MMCA/2015/43). Mice were euthanised by CO_2_ inhalation.

Human AGS gastric cancer cells stably expressing shRNA to *EGFP* (sh*EGFP*) or *NOD1* (sh*NOD1*) were generated by the integration of an expression vector containing a small interference RNA (siRNA) directed to the respective genes^25^. Human *NOD1* KO and control AGS cells were generated by CRISPR Cas/9 technology^17^. AGS and HEK293 epithelial cells were maintained in RPMI medium or DMEM, respectively, each supplemented with 10% (v/v) foetal calf serum (FCS), 1% (w/v) l-glutamine and 1% (w/v) penicillin/streptomycin (Gibco; ThermoFisher Scientific, Scoresby, VIC, Australia)^13^.

The mouse GSM06 GEC line (Riken Cell Bank RCB1779) was grown in Dulbecco’s modified Eagle medium–nutrient mixture/F-12 medium, supplemented with 10% FCS, 1% (w/v) insulin/transferring/selenite (Gibco) and 10 ng/ml epidermal growth factor^27^. Cells were grown in 5% CO_2_ at the permissive temperature of 33 °C, then moved to 37 °C prior to experiments.

*H. pylori* strains 251 (WT, Δ*cag*PAI, Δ*cagM*)^13,14^, 26695 (WT, *slt*^−^)^13^, 245m3^64^, B128 7.13^53^, the mouse-adapted SS1 strain and its clinical progenitor isolate 10700 (also known as PMSS1)^65^ were routinely grown on blood agar medium containing selective antibiotics at 37 °C under microaerobic conditions^27^. For MV preparation, *H. pylori* bacteria were grown in Brain Heart Infusion (BHI) broth (Oxoid; ThermoFisher Scientific) supplemented with 10% (v/v) heat-inactivated FCS (ThermoFisher Scientific) and antibiotics (BHIB medium), in a shaking incubator for 16–18 h at 37 °C under microaerobic conditions. MVs were isolated by ultracentrifugation of culture supernatants at 1,000,000 × *g* for 16 h^15^.

### Mouse *H. pylori* infection

Mice (6-8 week-old) were inoculated via oral gavage with 10^8^ colony-forming units of *H. pylori* strain SS1^27^, B128 7.13^53^, or 245m3^27^. Bacterial viability and numbers were determined by agar plate dilution. Stomachs and spleens were harvested at 1 and 8 weeks p.i. Gastric tissues were subjected to homogenisation using a gentleMACS™ Dissociator (Miltenyi Biotec, Macquarie Park, NSW, Australia). Bacterial infection status was confirmed by bacteriological culture^27^. Gastric homogenates were centrifuged at 13,000 × *g* at 4 °C and the supernatants were collected for analysis by the Qubit™ protein assay (ThermoFisher Scientific) and ELISA^66^.

BM reconstitution experiments were performed by subjecting recipient CD45.2 mice to two 5.5-Gy doses of irradiation given 3 hr apart and then injecting 1 × 10^6^ donor bone marrow cells via intravenous injection^60^. At 6 weeks post-transfer, recipient mice were infected with *H. pylori* SS1 and euthanised at 5 and 18 weeks p.i. The 5-week time-point was chosen to ensure full reconstitution of the immune system in animals. Reconstitution was confirmed by immunochemistry using an anti-CD45.1 antibody (dilution factor:1:100; clone A20, Biolegend, San Diego, CA, USA) (Supplementary Fig. 10).

### Inhibitor treatment

Cells were pre-treated with ML130 (5 μM; CID-1088438; Tocris, UK)^28^, WEHI-345 (0, 5 or 10 μM)^29^ or Z-YVAD-fmk (0, 1, 5, and 10 μM; 21874; Merck, Australia) for 1 h prior to stimulation with *H. pylori*, then maintained in the inhibitor-containing medium throughout the experiments.

### Histological analysis

Gastric tissues were formalin-fixed and embedded in paraffin. Tissues were stained with Mayer’s Haematoxylin & Eosin or Periodic acid-Schiff (PAS)–Alcian Blue (AB) stains and imaged at ×20 magnification using a bright-field microscope (Nikon Instruments Inc., NY, USA). Inflammatory scores for neutrophils and lymphocytes were graded in a blinded fashion (J.S.P.) according to a previously described grading scheme^27^. The mucosal thickness of the gastric corpus was determined in a blinded fashion (J.E.) on sections that had been digitised using an Aperio slide scanner (Leica Biosystems, Mount Waverley, VIC, Australia) and ImageScope software (Leica Biosystems). Three measurements were taken for each tissue section, with 3–4 sections available per stomach sample.

### Immunofluorescence

Antigen retrieval was performed by immersion in 10 mM sodium citrate buffer (pH 6) and boiling for 8 min in a microwave. Sections were washed twice with phosphate-buffered saline (PBS) and immersed in blocking buffer (5% horse serum, 1% Triton X-100). After 1 h, sections were incubated overnight at 4 °C with an antibody mix containing rat anti-mouse IL-18 (5 μg/ml; D047-3; R&D Systems, MN, USA) and rabbit anti-EpCAM (Clone ab71916; Abcam, Boston, MA, USA). The antibodies used in this study are listed in Supplementary Table 1. An isotype control for IL-18 staining was performed using mouse IgG (Dako, Santa Clara, CA, USA). Samples were washed thrice with blocking buffer and incubated for 2 h with anti-rat Alexa Fluor® 488- and anti-rabbit Alexa Fluor® 594-conjugated secondary antibodies (ThermoFisher Scientific, 1:400). After washing, cell nuclei were stained with Hoechst 33342 (ThermoFisher Scientific; 1:1000) for 5 min before mounting. Imaging was performed on a confocal microscope (Nikon). All images were captured using the same settings.

### Cytokine ELISA

Mouse IL-1β (BD Biosciences, North Ryde, NSW, Australia), IL-18 (R&D Systems, In Vitro Technologies Pty Ltd., VIC, Australia), Cxcl1/KC (R&D Systems), Cxcl2/MIP2 (R&D Systems), human IL-18 (R&D Systems) and CXCL8 (BD Biosciences) were quantified by ELISA, as per the manufacturers’ instructions. Production of bioactive IL-18 by AGS cells was also measured using the HEK-Blue™ IL-18 reporter cell line system (InvivoGen, San Diego, CA, USA).

### Cell sorting

Single-cell suspensions were prepared from the stomach tissues of *H. pylori*-infected mice. Briefly, whole stomachs were finely minced in disassociation buffer (2% FCS in Hank’s balanced salt solution without calcium and magnesium (Gibco; ThermoFisher Scientific) and 5 mM EDTA) and incubated at 37 °C with shaking at 180 rpm for 30 min. Tissues were passed through 70 μm cell strainers (Falcon; ThermoFisher Scientific) and the filtrates were collected. The remaining pieces of tissue were further digested at 37 °C with shaking at 180 rpm for 45 min in 2% FCS in RPMI (Life Technologies), containing 1 mg/ml collagenase Type 1 (Life Technologies), 0.4 units Dispase (Life Technologies) and 0.01 mg/ml DNase (Roche, North Ryde, NSW, Australia). Samples were passed through cell strainers (70 μm) and the filtrates were combined and centrifuged at 630 × *g* for 10 min at room temperature. Cell pellets were resuspended in FACS buffer (5% FCS in PBS, Life Technologies). After adding Fcγ receptor block (1:100 dilution; BD Biosciences) and incubating on ice for 10 min, dead cells were stained with PI (0.5 μg/ml; Life Technologies) and incubated for 30 min at 4 °C with antibodies specific for the following markers: CD45.2-PE-Cy7 (1:200 dilution; BD Biosciences); EpCAM-APC (1:100 dilution; eBioscience, CA, USA). Viable CD45.2^+^ EpCAM^−^ and CD45.2^−^EpCAM^+^ cells were sorted (FACS Aria Fusion; Beckman Coulter, VIC, Australia), verified for cell purity and the RNA isolated using Trizol (Life Technologies). Gating/sorting strategies are presented in Supplementary Fig. 11.

### Cell co-culture assays

*H. pylori* bacteria were grown in a BHIB medium, with shaking for 16–18 h under microaerobic conditions, at 37 °C^27^. Bacteria were pelleted and washed twice with PBS by centrifugation at 4000×*g* for 10 min at 4 °C prior to resuspension in RPMI medium for co-culture assays. Viable counts were performed by serial dilution of bacterial suspensions on blood agar or LB plates, respectively.

Cells were seeded in 12-well plates at 1 × 10^5^ cells/ml and allowed to grow overnight. The culture medium was removed and replaced with serum- and antibiotic-free media prior to stimulation with bacteria. In all cell co-culture experiments, *H. pylori* bacteria were added to cells at an MOI = 10:1^14^. The bacteria were removed after 1 h, and cells were washed and re-incubated at the appropriate times. Culture supernatants and lysates were collected at 24 h post-stimulation for ELISA and Western blot analyses. FLICA staining was performed as per the manufacturer’s instructions (ImmunoChemistry Technologies, MN, USA). The raw integrated density values for FLICA fluorescence were calculated by Fiji software after thresholding with the same thresholds applied to all images. The cell counter tool was used to count the number of nuclei and the ratio of FLICA fluorescence signal expressed per the total number of cells. Experiments were performed in triplicate. Cell death in AGS cells was assessed using the lactate dehydrogenase (LDH) assay. Cells were either treated with *H. pylori* for 1 h, washed and then left for 23 h, or treated with the pro-apoptotic drug, etoposide (50 µm), for 24 h. Cell proliferation was determined using the MTT assay (Promega Corp, Alexandria, VIC, Australia) on adherent AGS cells, while apoptosis was determined in AGS cells detached from culture plates by the addition of 0.25% Trypsin and 1 mM EDTA for 2 min. The isolated cells were subjected to Annexin V/PI (Life Technologies; Thermo Fisher Scientific) staining and analysed by flow cytometry using FlowJo™ software (vers. 10.5; BD Biosciences).

### Cell transfection

Specific silencer siRNAs for *TIFA* (40 nM; s40985)^15^ were diluted in Opti-MEM medium containing Lipofectamine® 2000 or RNAiMAX (all reagents from ThermoFisher Scientific), respectively, then incubated at room temperature for 20 min. The mixtures were added drop-wise to each well of 12-well plates containing AGS (10^5^) cells. As a control, cells were transfected with scramble siRNA (Silencer® Select Negative control No. 1 siRNA; ThermoFisher Scientific). Transfected cells were incubated at 37 °C for 24 h (*TIFA*), prior to co-culture with bacteria.

Cells were transfected with plasmids pSCFP3a-C1-NOD1^32^, pSCFP3a-C1-NOD1 K208R^32^, Caspase-1-GFP^67^, NOD1-mOrange^67^ or NOD1-YFP (also named TK71, generated in this work by inserting the full-length human *NOD1* gene into the EcoRI/BamHI sites of pEYFP-N1 vector), full-length NOD1-FLAG^67^ or ΔCARD-NOD1-FLAG^67^, diluted in Opti-MEM medium and supplemented with 2 μl Lipofectamine® 2000 reagent (ThermoFisher Scientific). Cells were then incubated at 37 °C for 24 h, prior to co-culture with bacteria. Co-localisation between NOD1 and caspase-1 was detected in sh*NOD1* AGS cells transfected with NOD1-YFP and stained with rabbit polyclonal anti-caspase-1 (AB_2068895; Santa Cruz Biotechnology, Texas, USA) and Alexa 488-conjugated anti-rabbit (Life Technologies) antibodies. Staining was visualised on a Nikon scanning confocal microscope. Co-localisation was analysed by Fiji software (version 1.0) and expressed by Pearson’s correlation coefficient.

### FLIM-FRET analysis

AGS cells were co-transfected with caspase-1-GFP (donor)^68^ and NOD1-mOrange^67^ (acceptor) before either *H. pylori* stimulation or control. FLIM data were recorded on an Olympus FV1000 confocal microscope equipped with a PicoHarp300 FLIM extension and 485 nm pulsed diode (PicoQuant, Germany). The pixel integration time for FLIM imaging was maintained at 40 µs/pixel and fluorescence lifetime histograms were accumulated to at least 20,000 counts to ensure sufficient data collection for FLIM-FRET analysis. Data was collected with photon count rates at 5% of the laser repetition rate to prevent pileup. FLIM-FRET analysis was performed using SymPhoTime 64 software (PicoQuant), with individual cells selected as regions of interest (ROIs) for analysis. In each experiment, ten random fields of view were recorded for each treatment and a minimum of five cells from each field were analysed. The fluorescence lifetime decay of each cell was deconvolved using the measured instrument response function (IRF) and then fitted with a bioexponential decay. The amplitude-weighted average lifetime was obtained from each fit and averaged over all cells for each condition.

### Western blot analyses

Cell culture supernatants were harvested and concentrated with StrataClean resin beads (Agilent Technologies, CA, USA), as per the manufacturer’s instructions. Cell lysates were prepared using NP-40 lysis buffer (ThermoFisher Scientific), supplemented with complete protease and phosphatase inhibitors (Roche). Total protein lysates (50 µg) were resuspended in Laemmli buffer (30 μl; ThermoFisher Scientific). All samples were heated at 98 °C for 10 min, loaded onto NuPAGE® 4–12% gels and run at 120 V in 1×MES buffer (ThermoFisher Scientific). The separated proteins were transferred onto membranes using the iBlot® transfer system (ThermoFisher Scientific), as per the manufacturer’s instructions. The membranes were blocked using Odyssey® blocking buffer (Odyssey; LI-COR, NE, USA). Membranes were incubated overnight at 4 °C, with rat anti-mouse IL-18 (1 μg/ml; D047-3; R&D Systems), rabbit anti-human IL-18 (0.2 μg/ml; sc-7954; Santa Cruz Biotechnology), mouse anti-human caspase-1 (0.1 μg/ml; sc56036; Santa Cruz Biotechnology), rabbit anti-ASC (1 μg/ml; clone AL177; AdipoGen, CA, USA), rabbit anti-human NLRP3 (1 μg/ml; Clone D2P5E; Cell Signalling, CA, USA) or mouse anti-FLAG (1 μg/ml; F1804; Sigma-Aldrich, Castle Hill, NSW, Australia). Membranes were washed in PBS-0.05% (v/v) Tween 20 and incubated for 2 h with goat-anti-mouse secondary antibody-Alexa Fluor® 680 conjugate or goat anti-rabbit secondary antibody-Alex Fluor® 800 (0.67 ng/ml; ThermoFisher Scientific). Membranes were washed and developed on the Odyssey Infra-red Imaging System (LI-COR). As loading controls, lysate samples were probed with rat-anti-human Tubulin (0.9 ng/ml; Rockland, PA, USA) and goat-anti-rat secondary antibody-Alexa Fluor® 800 conjugate (0.33 ng/ml; ThermoFisher Scientific). Protein transfer was confirmed by Ponceau S (Merck Life Science Pty Ltd, Bayswater, VIC, Australia) staining.

### Quantitative PCR (qPCR) analyses

RNA was extracted from cells using the PureLink® RNA mini kit (ThermoFisher Scientific). cDNA was generated from 500 μg of RNA using the Tetro cDNA synthesis kit (Bioline Australia, Eveleigh, NSW, Australia), as per the manufacturer’s instructions. cDNA (1 µl) was used per reaction, consisting of 5×Green GoTaq Flexi Buffer (Promega), dNTP mix (0.2 mM per dNTP) and primer (1 μM). Oligonucleotide sequences are presented in Supplementary Table 2. Denaturation was performed at 95 °C for 15 s, then 20 successive cycles of amplification (95 °C, 15 s; 60 °C, 1 min) and extension at 72 °C for 30 s. PCR products were run at 100 V for 20 min on 2% (w/v) agarose gels and stained with SYBR Safe DNA Gel Stain (Invitrogen; Carlsbad, CA, USA). Gels were visualised in a UV trans-illuminator and imaged using Quantum-Capture software.

For qPCR, reactions consisted of synthesised cDNA (4 μl, 1:10 dilution), SYBR® Green qPCR MasterMix (5 μl; ThermoFisher Scientific) and primer (1 µl, 1 μM). Assays were performed in an Applied Biosystems™ 7900 Fast Real-Time PCR machine (ThermoFisher Scientific), using the following programme: 50 °C, 2 min, followed by 95 °C, 10 min, then 40 successive cycles of amplification (95 °C, 15 s; 60 °C, 1 min). Gene expression levels were determined by the Delta-Delta Ct method using *RNA18S1* or *Rn18s* genes as references.

### Organoid cultures and microinjection

Gastric organoids were generated using an adaptation of previously described methods^69^. Briefly, mouse stomachs were dissected, cut open along the greater curvature, and washed thoroughly with PBS. The outer muscle layer was removed using fine-tipped forceps and the forestomach was discarded. The remaining tissue was cut into 2 mm pieces and washed in cold PBS before digestion with 10 mM EDTA for 4 h at 4 °C. To isolate glands, tissue fragments were vigorously suspended in cold PBS using 10 ml pipettes, and the supernatants were collected. This procedure was repeated a total of three times. Crypts were pelleted by centrifugation at 240 × *g* for 5 min, at 4 °C. Pellets were resuspended in PBS, passed through cell strainers (70 µm; Falcon) and centrifuged. Supernatants were discarded and the pellets containing the glands were resuspended in Matrigel (Corning; Optical Communications Pty Ltd, Mulgrave, VIC, Australia). Matrigel was seeded into each well of a 24-well plate (Nunc; Thermo Fisher Scientific) and incubated for 10 min at 37 °C until solidified. Crypt culture media (DMEM/F12 (Gibco), B27 (Gibco), Glutamax (Gibco), N2 (Gibco), 10 mM HEPES (Gibco), N-acetyl cysteine (1.25 µM; Sigma Aldrich), EGF (50 ng/ml; Peprotech, Lonza Australia, Mount Waverley, VIC, Australia), Noggin (100 ng/ml; Peprotech), Gastrin (10 nM; Sigma Aldrich), FGF10 (100 ng/ml; Peprotech), 10% (v/v) R-spondin 1-conditioned media, and 50% (v/v) Wnt3a-conditioned media) was added to each well. Established organoids were microinjected with 20 nl of overnight culture *H. pylori* 251 suspensions (10^7^ bacteria/ml) using the IM-300 pneumatic microinjector (Narishige; Carl Zeiss Pty Ltd, North Ryde, NSW, Australia). The presence of bacteria in the organoids was confirmed by immunofluorescence staining with rabbit anti-*H. pylori* antibody (in-house^15^) and anti-E-cadherin (5 μg/ml; clone DECMA-1, Abcam), followed by the appropriate secondary antibodies. Organoid viability was assessed using PrestoBlue reagent (Life Technologies; Thermo Fisher Scientific). Cell proliferation and apoptosis in the organoids were assessed using the MTT assay and Annexin V/PI straining, respectively.

### Statistical analyses

GraphPad Prism (GraphPad Software, CA, USA) was used for statistical analyses and graphical preparation. Data were analysed by the Student’s *T*-test (unpaired, two-tailed), one-way or two-way ANOVA (Tukey’s multiple comparisons test) or Kruskal–Wallis Test, as indicated. Differences were considered statistically significant for *p* values < 0.05.

### Reporting summary

Further information on research design is available in the Nature Portfolio Reporting Summary linked to this article.

## Supplementary information

Supplementary Information

Reporting Summary

## Data Availability

All data are included in the Supplemental Information or available from the authors upon reasonable request, as are unique reagents used in this Article. The raw numbers for charts and graphs are available in the Source Data file whenever possible. Source data are provided with this paper.

## Source data

Source Data
