# Supplementary material for: NOD1 mediates interleukin-18 processing in epithelial cells responding to Helicobacter pylori infection in mice
Source: Nat Commun. 2023 Jun 26;14:3804. doi: 10.1038/s41467-023-39487-1 (PMC10293252; doi:10.1038/s41467-023-39487-1)
Supplement: Supplementary file 1 — Supplementary Information [file 41467_2023_39487_MOESM1_ESM.pdf]

Supplementary Information

NOD1 mediates interleukin-18 processing in epithelial cells responding to *Helicobacter pylori* infection in mice.

First author: L. S. Tran

Corresponding author: Richard L. Ferrero

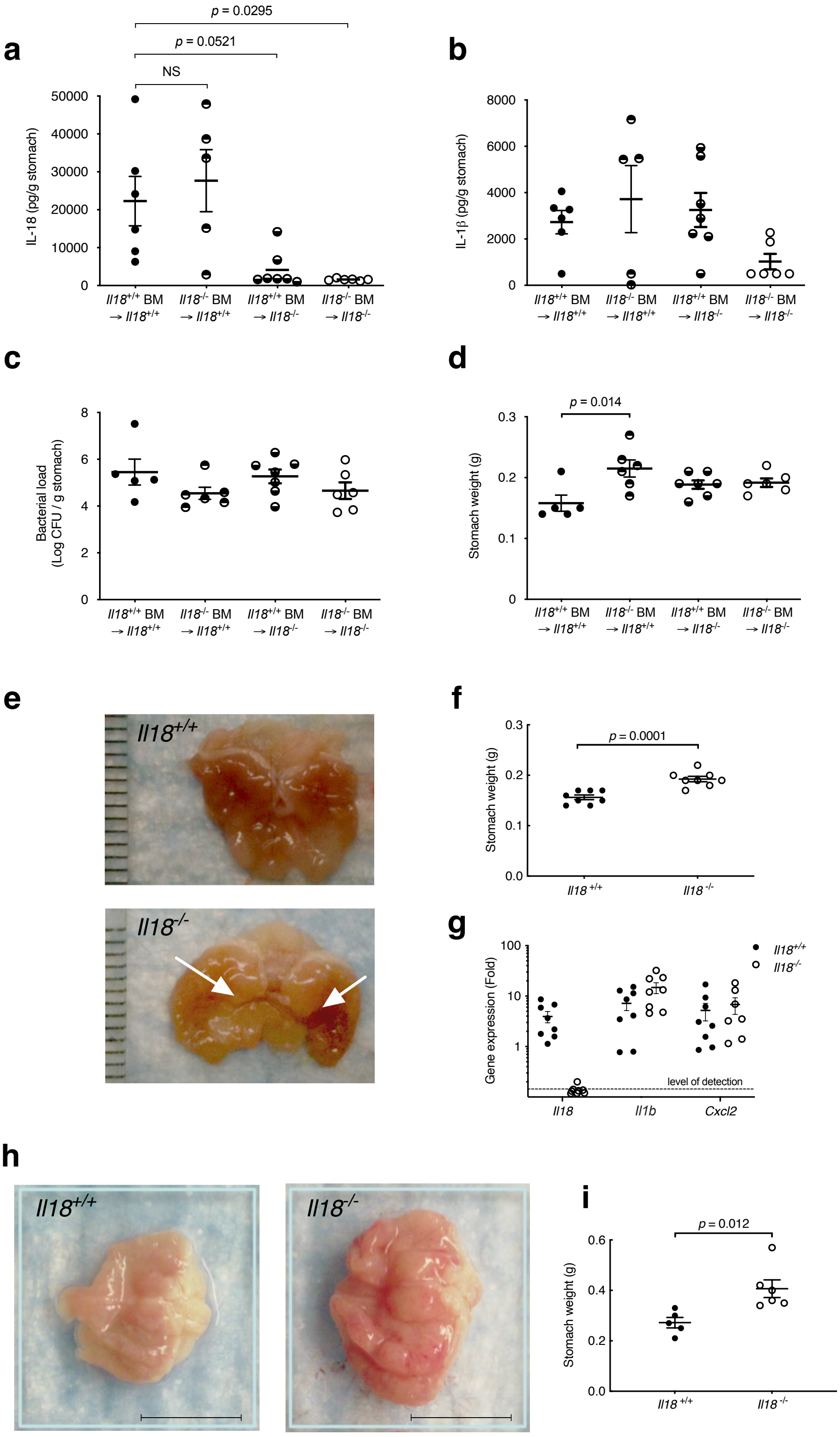

Supplementary Fig. 1 IL-18 plays a protective role against *Helicobacter*-induced pre-neoplastic lesions in the stomach. **a-d** Bone marrow (BM) reconstitution experiments were performed by transferring BM from either *Il18*$^{+/+}$ or *Il18*$^{-/-}$ donor mice to γ-irradiated *Il18*$^{+/+}$ or *Il18*$^{-/-}$ recipient mice

(n=24 females). Mice were then challenged with *H. pylori* SS1 and culled at 18 weeks p.i. **a** IL-18 and **b** IL-β levels in stomach homogenates. **c** *H. pylori* bacterial loads. **d** Stomach weights. **e-i** *Il18*$^{+/+}$ and *Il18*$^{-/-}$ mice at either **e-g** 8weeks p.i. with *H. pylori* SS1 (n=8/group; 10 males, 6 females) or **h, i** 52 weeks p.i. with *H. felis* (11 females). **e, h** Gross pathology of stomachs with arrows indicating thickening of the mucosa in an *Il18*$^{-/-}$ animal. **f, h** Stomach weights. **g** Cytokine and chemokine gene expression in mouse stomachs. Scale bars, 1mm (**e**) or 1 cm (**h**). Data correspond to the mean ± SEM, with each data point representing an individual mouse. Significance was determined by one-way ANOVA (**a-d**) or two-sided Student's t-test (**f, i**).

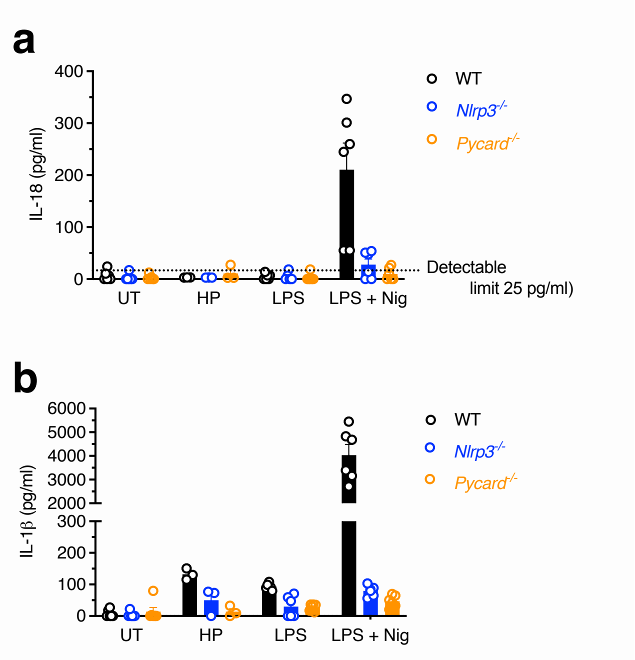

Supplementary Fig. 2 BMDMs do not produce IL-18 in response to *H. pylori* stimulation. **a-b** IL-18 (**a**) and IL-1β (**b**) production in BMDMs from wild type (WT), *Nlrp3*$^{-/-}$ and *Pycard*$^{-/-}$ mice. BMDMs were either left untreated (UT) or stimulated with *H. pylori* bacteria (HP), *E. coli* LPS (LPS) alone or with the inflammasome activator, nigericin (LPS + Nig). Mean ± SEM for combined data from two independent experiments with duplicate and triplicate replicates shown for HP-treated and UT, LPS- or LPS + Nig-stimulated samples, respectively.

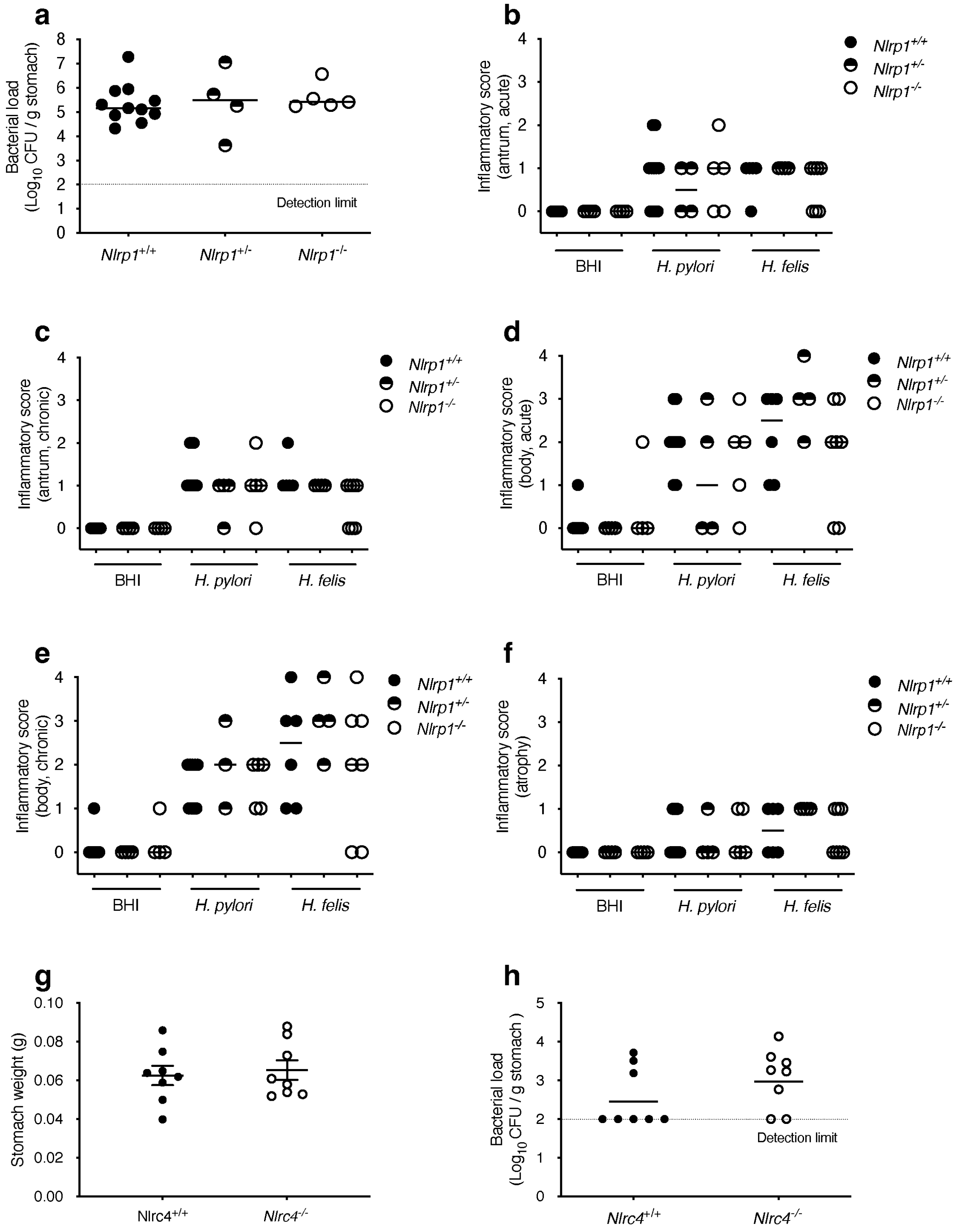

Supplementary Fig. 3 Mice lacking the canonical inflammasome proteins Nlrp1 and Nlrc4 are unaffected in *H. pylori* colonisation and pathology. **a-f** *Nlrp*$^{+/+}$, *Nlrp1*$^{+/-}$, *Nlrp*$^{-/-}$ and **g, h** *Nlrc4*$^{+/+}$ and *Nlrc4*$^{-/-}$ mice were administered either BHI broth (control; **a-f**), *H. pylori* SS1 (**a-f**), *H. pylori* B128

7.13 (**g, h**) or *H. felis* (**a-f**). Mice were culled at 12 weeks (**a**-**f**) and 2 weeks (**g, h**) p.i. Stomachs from the mice were assessed for *H. pylori* bacterial loads (**a, h**), inflammation (**b-f**) and weights (**g**). Mouse numbers and sexes were as follows: *Nlrp1*$^{+/+}$ (13 males, 15 females), *Nlrp1*$^{+/-}$ (6 males, 5 females), *Nlrp*$^{-/-}$ (8 males, 8 females), *Nlrc4*$^{+/+}$ (3 males, 5 females) and *Nlrc4*$^{-/-}$ (8 females). Combined data from two independent experiments (**g, h**). Data correspond to the mean (**a-f, h**) and mean ± SEM (**g**), with each data point representing an individual mouse.

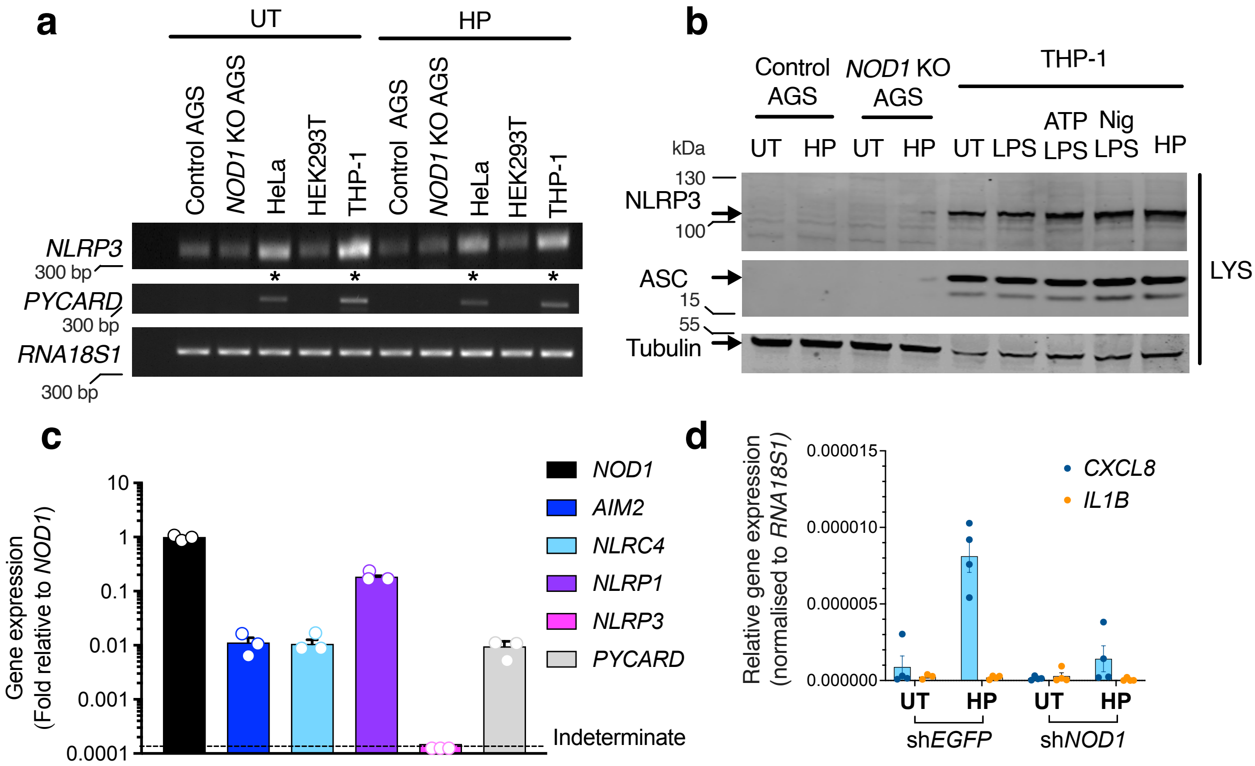

Supplementary Fig. 4 Human AGS gastric epithelial cells do not express detectable levels of the canonical inflammasome proteins, NLRP3 or ASC. RT-PCR detection of *NLRP3* and *PYCARD* (**a**) expression in control AGS and *NOD1* KO CRISPR/Cas9 cell lines, as well as in control cell lines HeLa, HEK293T and THP-1. Cells were either left untreated (UT) or stimulated with *H. pylori* bacteria (HP). *RNA18S1* expression was determined as a loading control. *, indicates positive *NLRP3* and *PYCARD* PCR results. **b** Western blot analysis of NLRP3 and ASC synthesis in cell lysates (LYS). Tubulin was used as a loading control. Detection of NLRP3 and ASC proteins was performed on the same gel/blot, whereas tubulin was detected on a different gel/blot. **c** qPCR detection of gene expression for select NLR and inflammasome proteins in AGS cells under basal conditions. **d** *CXCL8* and *IL1B* gene expression in sh*EGFP* and sh*NOD1* AGS cells that were either left untreated (UT) or stimulated with *H. pylori* bacteria (HP). Mean ± SEM, with triplicate (**c**) or quadruplicate (**d**) replicates.

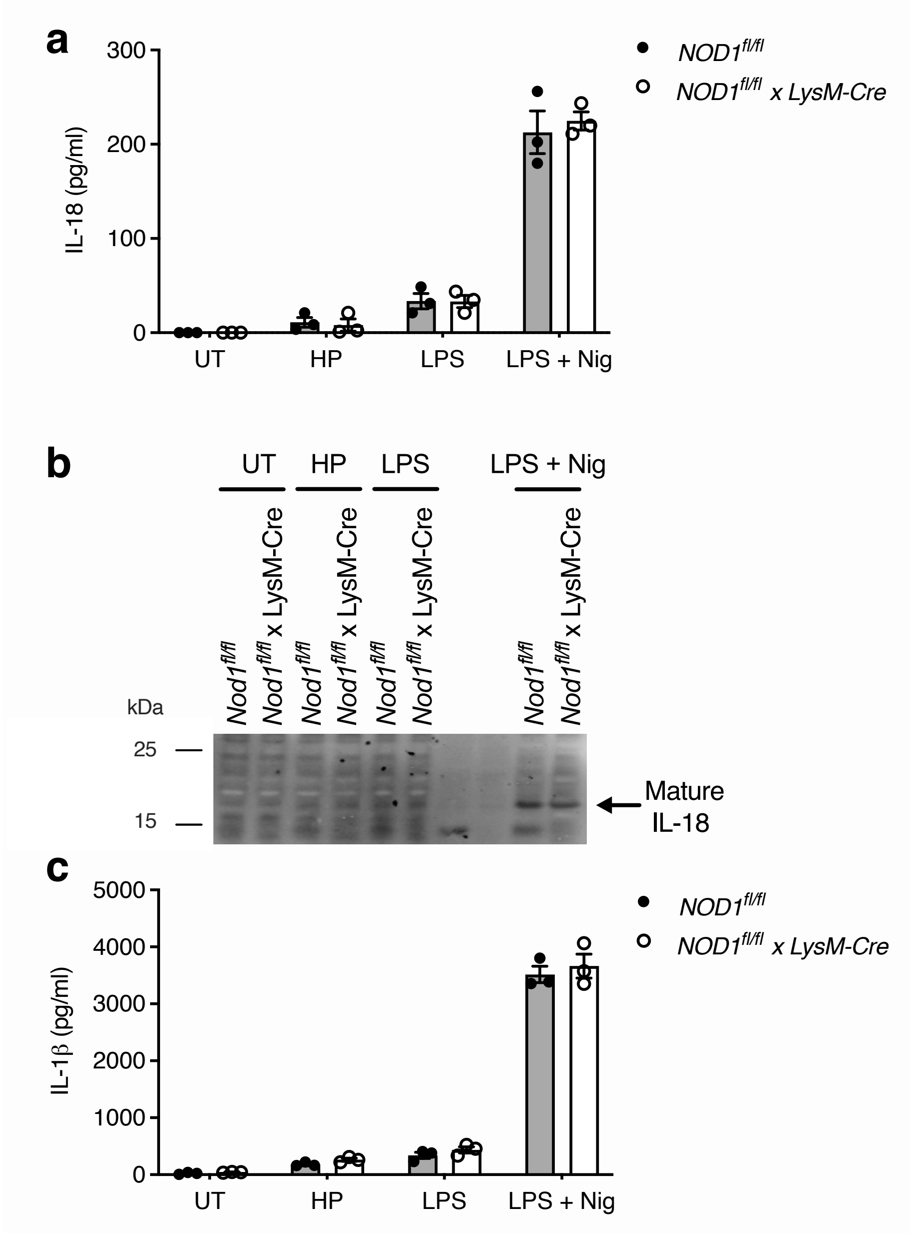

Supplementary Fig. 5 Nod1 deficiency has no effect on IL-18 production by BMDMs. **a-c** Total IL-18 (**a**), mature IL-18 (**c**) and IL-1β (**c**) production by BMDMs from *Nod1*$^{fl/fl}$ and *Nod1*$^{fl/fl}$ x *LysM*-cre mice. BMDMs were either left untreated (UT) or stimulated with *H. pylori* bacteria (HP), *E. coli* LPS (LPS) alone or with the inflammasome activator, nigericin (LPS+Nig). Three independent experiments. Representative figure (**b**), or mean ± SEM and triplicate replicates (**a, c**).

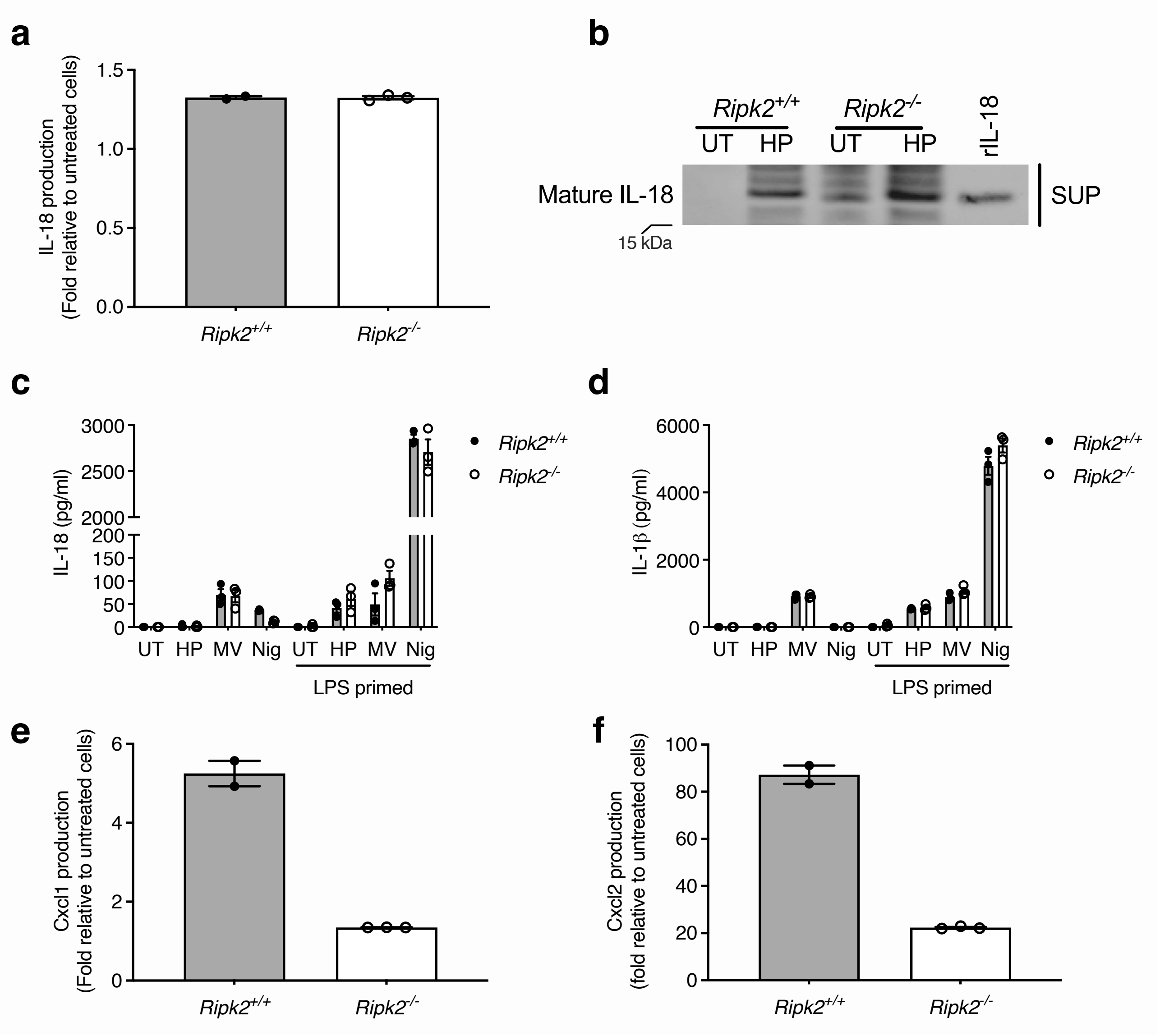

Supplementary Fig. 6 Ripk2 deficiency has no effect on IL-18 production. **a-b** Total IL-18 (**a**), mature IL-18 (**b**) production by GECs isolated from *Ripk2*$^{+/+}$ and *Ripk2*$^{-/-}$ mice. **c-d** Total IL-18 (**c**) and IL-1β (**d**) production by BMDMs isolated from *Ripk2*$^{+/+}$ and *Ripk2*$^{-/-}$ mice. BMDMs were either left untreated (UT) or stimulated with *H. pylori* bacteria (HP), HP outer membrane vesicles (MV), *E. coli* LPS (LPS) or LPS together with the inflammasome activator, nigericin. **e**, **f** Fold changes of Cxcl1 (**e**) and Cxcl2 (**f**) production by HP-stimulated BMDMs from *Ripk2*$^{+/+}$ and *Ripk2*$^{-/-}$ mice normalised to the corresponding untreated cells. Two independent experiments. Representative image (**b**) and mean ± SEM with duplicate (**a, e, f**) or triplicate (**a, c- f**) replicates shown.

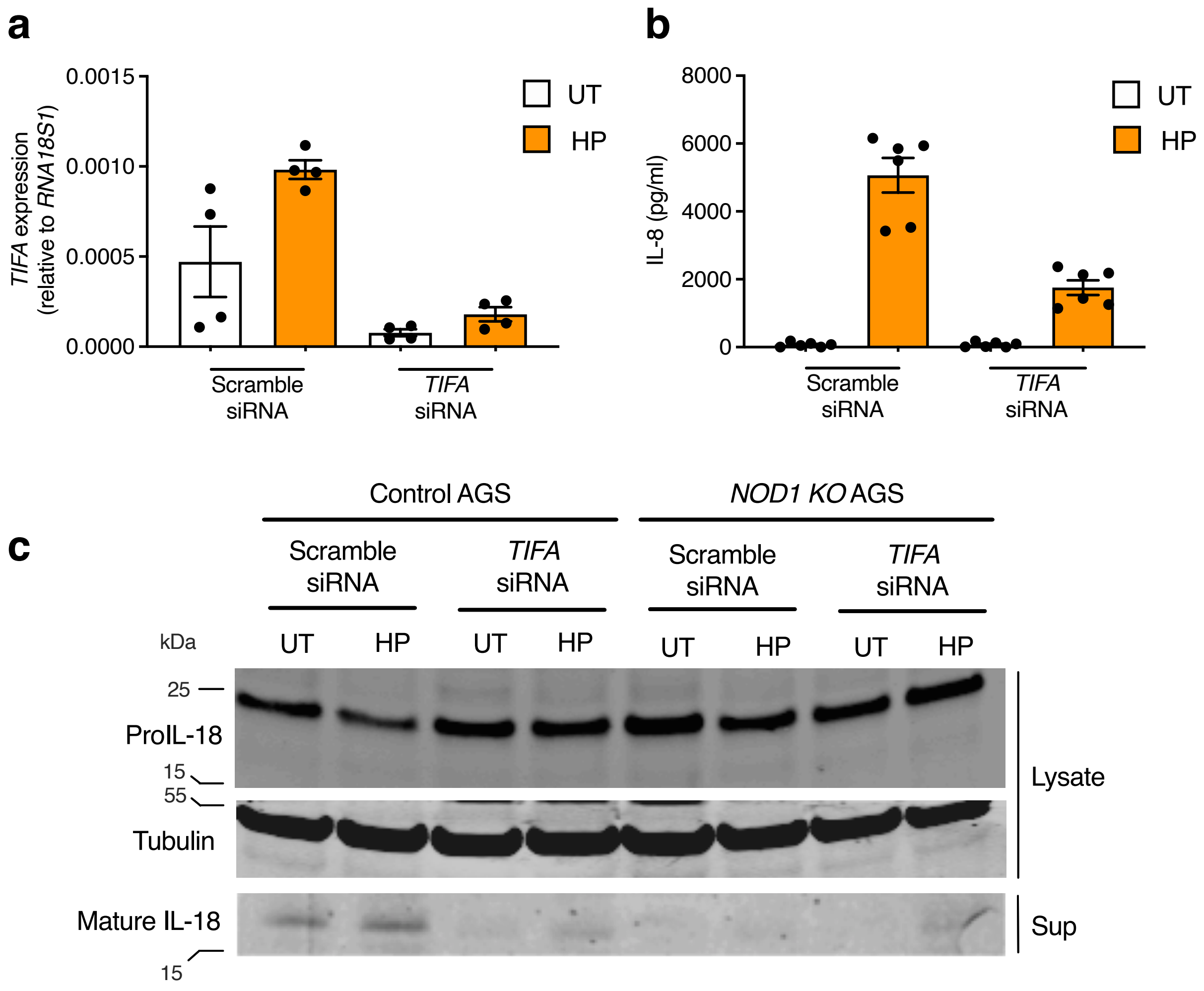

Supplementary Fig. 7 TIFA is involved in the IL-18 processing induced by *H. pylori* in gastric epithelial cells. AGS control (**a-c**) or NOD1 KO (**c**) cells were transfected with scramble or *TIFA* siRNA then either left untreated (UT) or stimulated with *H. pylori* (HP). *TIFA* KD was confirmed by qPCR (**a**), while IL-8 production (**b**) and IL-18 processing (**c**) were assessed in culture supernatants. Representative data (**a, b**) and image (**c**) of three independent experiments. Mean ± SEM for combined data from two experiments with duplicate (**a**) and triplicate (**b**) replicates shown.

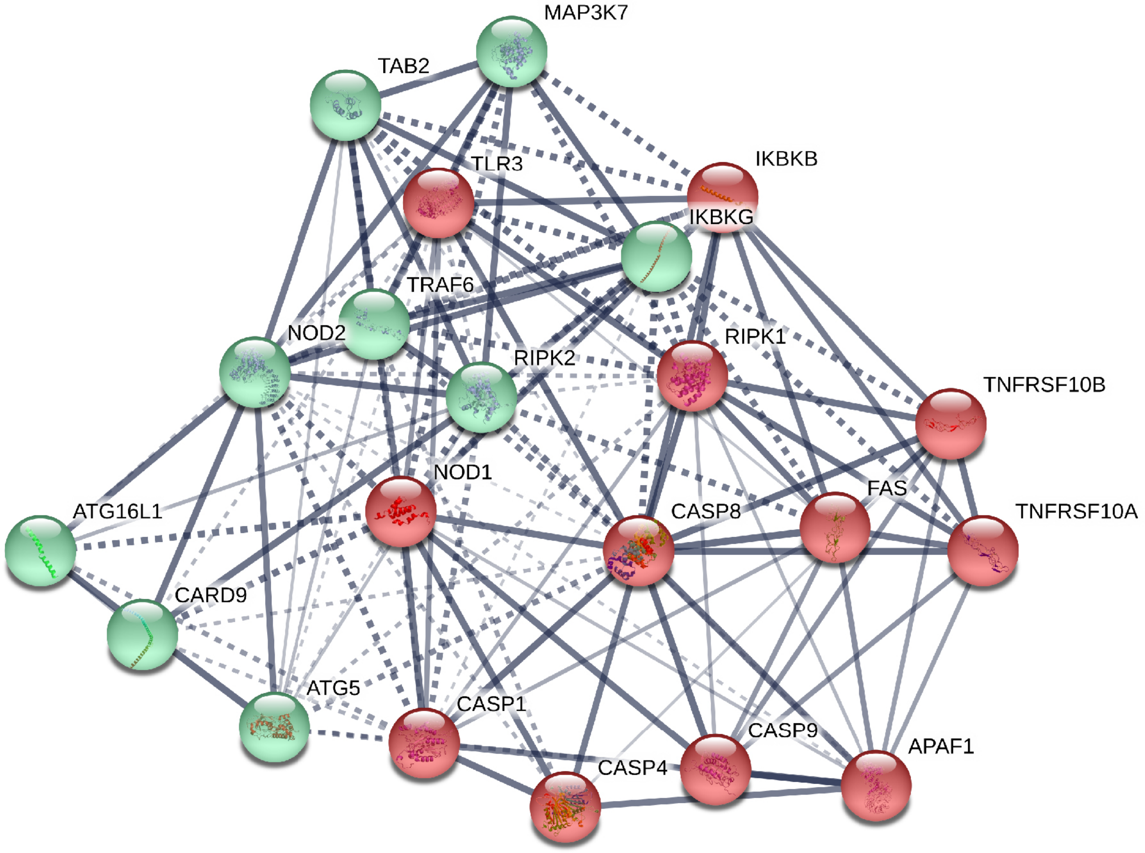

Supplementary Fig. 8 Protein-protein interaction network for human NOD1, as visualised by STRING. Two major protein interaction nodes, indicated in red and green, were identified by *k*-means clustering. The thickness of the lines represents the confidence score of the functional associations between proteins.

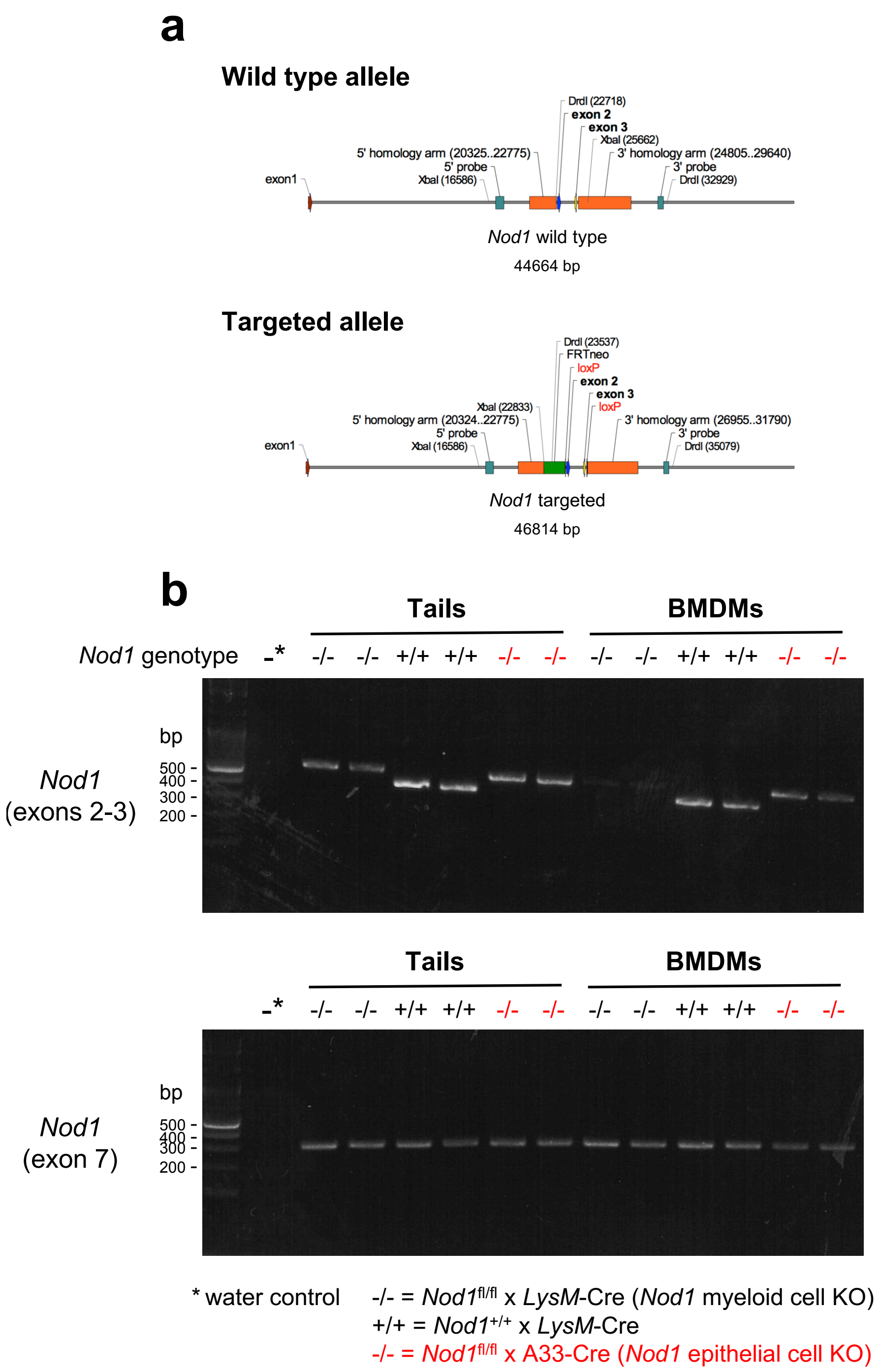

Supplementary Fig. 9 Generation of conditional knockout mice lacking *Nod1*$^{-/-}$ in the myeloid compartment. **a** C57BL/6 mice were generated in which the *Nod1* gene was "floxed" using a gene targeting strategy that involved deletion of *Nod1* exons 2 and 3, corresponding to the *Nod1* CARD [1]. For this, a construct was generated in which *loxP* sites were situated external to these two exons and an FRT-flanked neomycin cassette used for selection. A detailed description of the gene targeting strategy used to select for embryonic stem cells carrying the "floxed" allele and generation of the

gene targeted mice is described in Pusceddu *et al.* [1]. To generate mice lacking functional Nod1 in the myeloid compartment, *Nod1*$^{fl/fl}$ mice were crossed with *lysM*-Cre animals [2]. **b** *Nod1* PCR was performed on tail DNA and BMDM cDNA from two of these mice, using the following oligonucleotide pairs, specific for exons 2-3 and 7, respectively: CTTATCTTTGTTCACATAGCAACC and GATCCTGCCTTTCATTGCTGACC; GGAGGCCAACAGACGCCT and ACTGACCTAGAGGGTATCG. DNA/cDNA samples were also tested from the progeny of *Nod1*$^{fl/fl}$ mice crossed with epithelial cell-specific Cre (A33-Cre) animals (https://pb.apf.edu.au/phenbank/strain.html?id=5502) and from *Nod1*$^{+/+}$ mice crossed with *lysM*-Cre animals. Wild type and floxed *Nod1* are identified by 432- and 551-base pair (bp) PCR products, respectively. Water was used as a negative control.

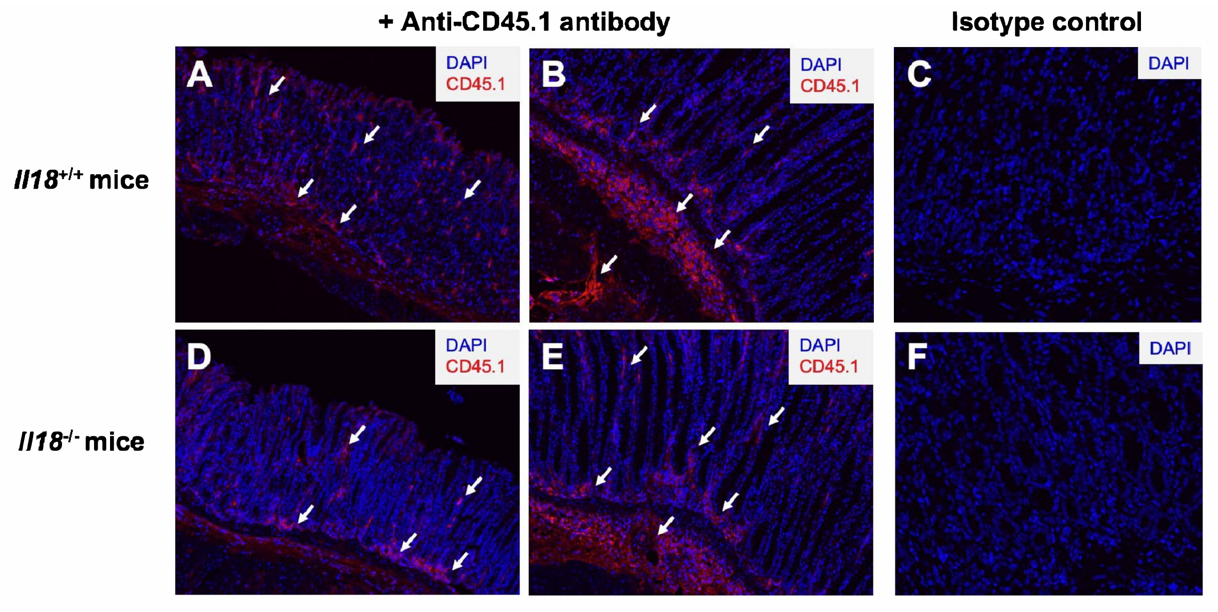

Supplementary Fig. 10 Confirmation of bone marrow (BM) reconstitution in mice by immunohistochemical detection of CD45.1 cells in the gastric mucosa of CD45.2 mice. Representative images showing the gastric tissues of *Il18*$^{+/+}$ **(A-C)** and *Il18*$^{-/-}$ **(D-F)** mice reconstituted with BM from CD45.1 animals. Tissues were reacted with either anti-CD45.1 **(A, B, D, E)** or isotype control **(C, F)** antibodies. Arrows indicate CD45.1^{+} cells in the lamina propria and submucosa of tissues.

Gating strategy for sorting EpCAM+ epithelial cells and CD45+ immune cells (Figure 1c)

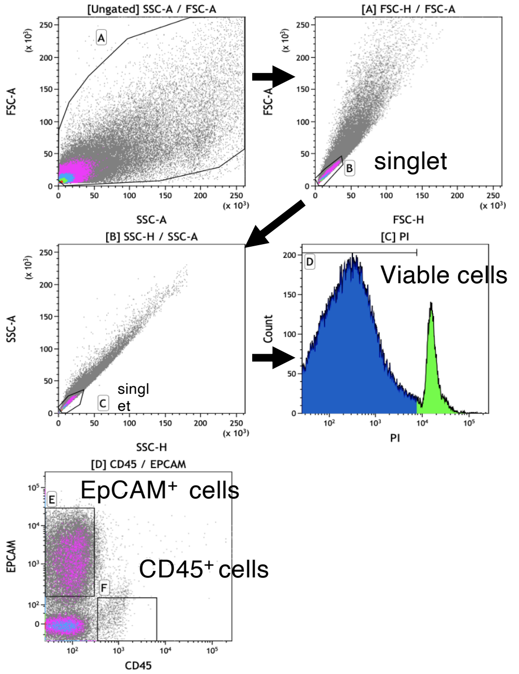

Annexin V/PI gating strategy for AGS cells (Figure 8b)

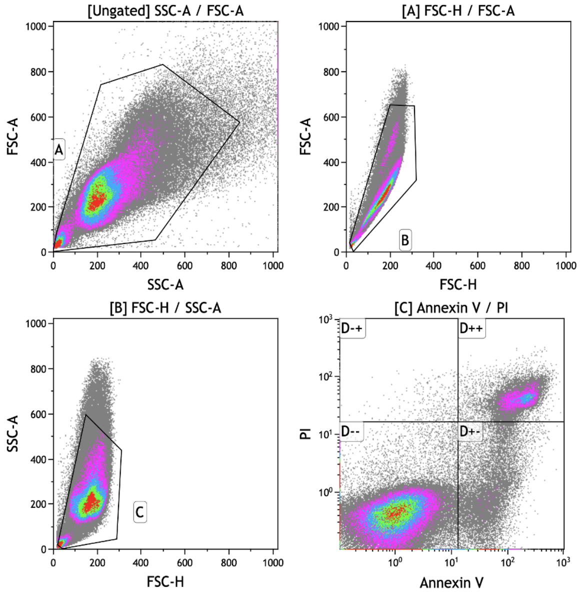

Annexin V/PI gating strategy for organoids (Fig. 8e)

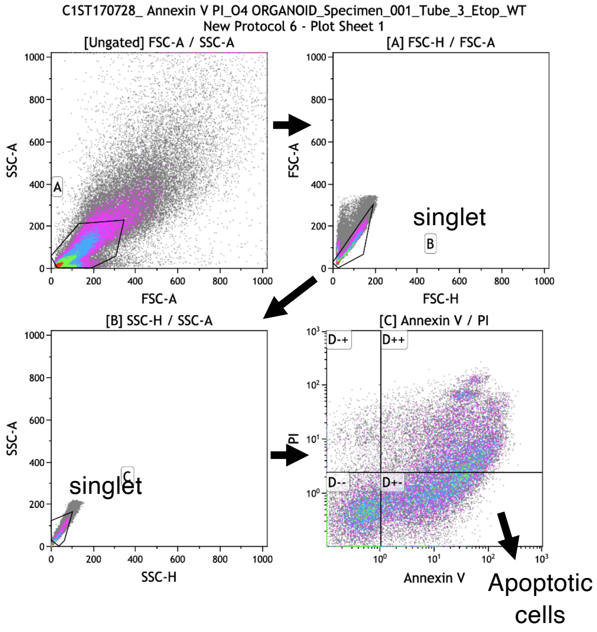

Supplementary Figure 11. The gaiting steps for each sorting strategy used in the study. Single-cell suspensions were prepared from stomach tissues of *H. pylori*-infected mice using the protocol detailed in the Materials and Methods section. Briefly, cells were incubated with the appropriate antibodies, stained with a viability dye (PI) and resuspended in 1X PBS-5% FBS for sorting. AGS

cells were harvested from culture plates using 0.25% Trypsin and 1 mM EDTA, then immediately stained with PI and Annexin V. For organoids, single cells were harvested from Matrigel-coated plates by incubation in 300 µl TrypLE express (ThermoFisher Scientific, Scoresby, VIC, Australia) at 37°C for 30 min. Cells were separated by pipetting up and down with 1 ml pipet tips. Single cells were stained with PI and Annexin V. Cell sorting was performed using a FACS Aria sorter and FACS Canto Analyzer (BD Biosciences, North Ryde, NSW, Australia). Data were analysed using Kaluza software (Beckman Coulter). To identify events corresponding to cells, all samples were initially gated using forward- and side-scatter. Single cells were then enriched by gating for forward scatter height vs. area. Viable cells were selected by negativity using PI. Cell purity was determined after completion of the sort.

Supplementary Table 1. Antibodies used in the Study.

Host Species	Species Reactivity	Target (label)	Conc. or Dilution	Catalogue no. and/or Clone and Manufacturer
Rat	M	IL-18	1-5 µg/ml	Cat. no. D047-3; R&D Systems, MN, USA
Rat	H	IL-18	0.2 µg/ml	Cat. no. sc-7954; Santa Cruz Biotechnology, TX, USA
Rabbit	M, R, H[a]	EpCAM	1:100	Clone ab71916; Abcam, MA, USA
Rat	H, M	EpCAM-APC	1:100	Clone G8.8; eBioscience, CA, USA
Mouse	M	CD45.1	1:100	Clone A20, cat no. 110702; Biolegend, CA, USA
Mouse	M	CD45.2-PE-Cy7	1:200	Clone 104; BD Biosciences, VIC, Australia
Rabbit	M, R	Caspase-1	1:100	Cat. no. AB_2068895; Santa Cruz Biotechnology, TX, USA
Mouse	H	Caspase-1	0.1 µg/ml	Cat. no. sc56036; Santa Cruz Biotechnology, TX, USA
Rabbit	H, M	ASC	1 µg/ml	Clone AL177; AdipoGen, CA, USA
Rabbit	H	NLRP3	1 µg/ml	Clone D2P5E; Cell Signaling, CA, USA
Mouse	Not applicable	FLAG	1 µg/ml	Cat. no. F1804; Sigma-Aldrich, NSW, Australia
Rabbit	H, M, R	Tubulin	0.9 ng/ml	Cat. no. F1804600-401-880; Rockland, PA, USA
Rabbit	Not applicable	*H. pylori* whole bacteria	1:1,000	"In-house" [3]

Rat	M	E-cadherin	5 µg/ml	Clone DECMA-1; Abcam, MA, USA
Rabbit	H, M, R	NOD1	1:100	Cat. no. 3545; Cell Signaling Technology, MA, USA
Rabbit	R	Alexa Fluor® 488	1:400	Cat. no. A-21210; Thermo Fisher Scientific, VIC, AUS
Goat	Rabb	Alexa Fluor® 594	1:400	Cat. no. A-11012; Thermo Fisher Scientific, VIC, AUS
Goat	Rabb	Alex Fluor® 800	0.33-0.67 ng/ml	Cat. no. A32735; Thermo Fisher Scientific, VIC, AUS

[a] M = mouse, H = human, Rabb = rabbit, R = rat

Supplementary Table 2. Sequences of oligonucleotides used in this Study.

**Target genes**	**Oligonucleotide sequences**	
RNA18S1	Fwd-CGGCTACCACATCCAAGGAA	Rev-GCTGGAATTACCGCGGCT
AIM2	Fwd-TGAAACCCCGAAGATCAACAC	Rev-CCCAGTACTTCCATTTTCCCAG
PYCARD	Fwd-TCACCGCTAACGTGCTG	Rev-TGGTCTATAAAGTGCAGGCC
NOD1	Fwd-ACGATGAAGTGGCAGAGAGTT	Rev-GGCAGTCCCCTTAGCTGTGA
NLRC4	Fwd-CAGTCCCCTCACCATAGAAG	Rev-TCAAGTTACCCAAGCTGTCAG
NLRP1	Fwd-GGACTGACGATGACTTCTGG	Rev-ATCACAAAGCAGAGACCCG
NLRP3	Fwd-GTGTTTCGAATCCCACTGTG	Rev-TCTGCTTCTCACGTACTTTCTG
RIPK2	Fwd-GCCACCTGAAAACTATGAACC	Rev-CTGCAAAGGATTGGTGACATC
TIFA	Fwd-TCGATTCCCCTCGCTCTG	Rev-CCGTCATCTGGAGACAAGTTAC
Rn18s	Fwd-GTAACCCGTTGAACCCCATT	Rev-CCATCCAATCGGTAGTAGCG
Cxcl2	Fwd-AACATCCAGAGCTTGAGTGTGA	Rev-TTCAGGGTCAAGGCAAACTT
Il1b	Fwd-ACGGACCCCAAAAGATGAAG	Rev-TTCTCCACAGCCACAATGAG
Il18	Fwd-GCCTCAAACCTTCCAAATCAC	Rev-GTTGTCTGATTCCAGGTCTCC
Nod1	Fwd-GGAGGCCAACAGACGCCT	Rev-ACTGACCTAGAGGGTATCG

References

1. Pusceddu, M. M. *et al.* Nod-like receptors are critical for gut-brain axis signalling in mice. *J Physiol* **597**, 5777-5797 (2019).

2. Chonwerawong, M. *et al.* Innate immune molecule NLRC5 protects mice from *Helicobacter*-induced formation of gastric lymphoid tissue. *Gastroenterol* **159**, 169-182 (2020).

3. Kaparakis M*, et al.* Bacterial membrane vesicles deliver peptidoglycan to NOD1 in epithelial cells. *Cell Microbiol* **12**, 372-385 (2010).
