# Supplementary material for: NOD1 mediates interleukin-18 processing in epithelial cells responding to Helicobacter pylori infection in mice
Source: Nat Commun. 2023 Jun 26;14:3804. doi: 10.1038/s41467-023-39487-1 (PMC10293252; doi:10.1038/s41467-023-39487-1)
Supplement: Supplementary file 2 — Reporting Summary [file 41467_2023_39487_MOESM2_ESM.pdf]

Corresponding author(s): Richard L. Ferrero

Last updated by author(s): 23-05-26

Reporting Summary

Nature Portfolio wishes to improve the reproducibility of the work that we publish. This form provides structure for consistency and transparency in reporting. For further information on Nature Portfolio policies, see our Editorial Policies and the Editorial Policy Checklist.

Statistics

For all statistical analyses, confirm that the following items are present in the figure legend, table legend, main text, or Methods section.

n/a	Confirmed	
☐	☒	The exact sample size (*n*) for each experimental group/condition, given as a discrete number and unit of measurement
☐	☒	A statement on whether measurements were taken from distinct samples or whether the same sample was measured repeatedly
☐	☒	The statistical test(s) used AND whether they are one- or two-sided<br>*Only common tests should be described solely by name; describe more complex techniques in the Methods section.*
☒	☐	A description of all covariates tested
☒	☐	A description of any assumptions or corrections, such as tests of normality and adjustment for multiple comparisons
☐	☒	A full description of the statistical parameters including central tendency (e.g. means) or other basic estimates (e.g. regression coefficient) AND variation (e.g. standard deviation) or associated estimates of uncertainty (e.g. confidence intervals)
☐	☒	For null hypothesis testing, the test statistic (e.g. *F*, *t*, *r*) with confidence intervals, effect sizes, degrees of freedom and *P* value noted<br>*Give P values as exact values whenever suitable.*
☒	☐	For Bayesian analysis, information on the choice of priors and Markov chain Monte Carlo settings
☒	☐	For hierarchical and complex designs, identification of the appropriate level for tests and full reporting of outcomes
☐	☒	Estimates of effect sizes (e.g. Cohen's *d*, Pearson's *r*), indicating how they were calculated

Our web collection on statistics for biologists contains articles on many of the points above.

Software and code

Policy information about availability of computer code

Data collection	Quantum-Capture (v2022.07.19), ImageScope (v.12.1)
Data analysis	Fiji (vers. 1.0), Flojo (vers. 10.5), SymPhoTime 64 (PicoQuant; vers. 2.7), GraphPad (Prism; vers. 9.3.1)

For manuscripts utilizing custom algorithms or software that are central to the research but not yet described in published literature, software must be made available to editors and reviewers. We strongly encourage code deposition in a community repository (e.g. GitHub). See the Nature Portfolio guidelines for submitting code & software for further information.

Data

Policy information about availability of data

All manuscripts must include a data availability statement. This statement should provide the following information, where applicable:

- Accession codes, unique identifiers, or web links for publicly available datasets
- A description of any restrictions on data availability
- For clinical datasets or third party data, please ensure that the statement adheres to our policy

The authors declare that all data supporting the findings of the study are available in this article and its supplementary information files. The source data for Figures and Supplementary Figures in this study are provided in the Source Data file.

Human research participants

Policy information about studies involving human research participants and Sex and Gender in Research.

Reporting on sex and gender	Not applicable
Population characteristics	Not applicable
Recruitment	Not applicable
Ethics oversight	Not applicable

Note that full information on the approval of the study protocol must also be provided in the manuscript.

Field-specific reporting

Please select the one below that is the best fit for your research. If you are not sure, read the appropriate sections before making your selection.

☒ Life sciences ☐ Behavioural & social sciences ☐ Ecological, evolutionary & environmental sciences

For a reference copy of the document with all sections, see nature.com/documents/nr-reporting-summary-flat.pdf

Life sciences study design

All studies must disclose on these points even when the disclosure is negative.

Sample size	Sample sizes for in vivo experiments were based on our previous experimental studies in mouse Helicobacter infection models in: Ferrero et al.(1997). "Local immunoglobulin G antibodies in the stomach may contribute to immunity against Helicobacter infection in mice." Gastroenterol. 113, 185-194; Ferrero, et al. (1998). "Immune responses of specific-pathogen-free mice to chronic Helicobacter pylori (strain SS1) infection." Infect. Immun. 66, 1349-1355; Viala et al. (2004) "Nod1 responds to peptidoglycan delivered by the Helicobacter pylori cag pathogenicity island." Nature Immunol. 5, 1166-1174.) Based on power calculations from these studies, sample sizes of $n \geq 8$ mice per group are ideal but do depend on the phenotype under investigation and not always possible when breeding several lines of knockout mice. In the few instances when these numbers were not attained in the current study, we were clearly able to establish biological significance or otherwise. To further improve statistical significance in some infeciton studies, data were combined from 2 independent experiments.<br>For in vitro experiments, no statistical methods were used. Sample size was determined based on standards for cell biology experiments, particularly our own previous studies in Helicobacter co-culture models, as follows: Gobert et al. (2004). "Helicobacter pylori Heat Shock Protein 60 mediates interleukin-6 production by macrophages via a Toll-like Receptor (TLR)-2-, TLR-4- and Myeloid Differentiation factor 88-independent mechanism." J. Biol. Chem. 279, 245-250; Viala et al. (2004) "Nod1 responds to peptidoglycan delivered by the Helicobacter pylori cag pathogenicity island." Nature Immunol. 5, 1166-1174; Allison et al. (2009) "Helicobacter pylori induces MAPK phosphorylation and AP-1 activation via a NOD1-dependent mechanism." J Immunol. 183: 8099-8109; Kaparakis et al. (2010) "Bacterial membrane vesicles deliver peptidoglycan to NOD1 in epithelial cells." Cell Microbiol. 12, 372-385. Generally, a sample number of three independent experiments was used but fewer numbers were used for primary gastric epithelial cells or imaging studies involving the analysis of multiple fields of view.
Data exclusions	No data were excluded from the current version of the manuscript.
Replication	Results were verified by independent biological and/or technical replicates, as reported in the Figure legends, thus confirming successful data replication.
Randomization	For mouse experimentation, animals in each cage were randomly assigned to different groups. Randomisation was not required for the in vitro experiments as all measurements were performed in parallel. For image analysis (Fig. 6a-d), fields were chosen randomly and data analysed unbiasedly using software.
Blinding	All histopathological analyses were performed in a blinded fashion.<br>For in vitro experiments, blinding was not relevant because it would not affect the quantitative results e.g. IL-8 production, IL-18 synthesis and processing, co-localisation of signalling molecules, cell apoptosis or proliferation.

Reporting for specific materials, systems and methods

We require information from authors about some types of materials, experimental systems and methods used in many studies. Here, indicate whether each material, system or method listed is relevant to your study. If you are not sure if a list item applies to your research, read the appropriate section before selecting a response.

Materials & experimental systems

n/a	Involved in the study
☐	☒ Antibodies
☐	☒ Eukaryotic cell lines
☒	☐ Palaeontology and archaeology
☐	☒ Animals and other organisms
☒	☐ Clinical data
☒	☐ Dual use research of concern

Methods

n/a	Involved in the study
☒	☐ ChIP-seq
☐	☒ Flow cytometry
☒	☐ MRI-based neuroimaging

Antibodies

Antibodies used

Host Species	Species Reactivity	Target (label)	Conc. or Dilution	Catalogue no. and/or Clone and Manufacturer
1) Rat	Mouse (M)	IL-18	1-5 µg/ml	Cat. no. D047-3; R&D Systems, MN, USA
2) Rat	Human (H)	IL-18	0.2 µg/ml	Cat. no. sc-7954; Santa Cruz Biotechnology, TX, USA
3) Rabbit	M, Rat (R), H	EpCAM	1:100	Clone ab71916; Abcam, MA, USA
4) Rat	H, M	EpCAM-APC	1:100	Clone G8.8; eBioscience, CA, USA
5) Mouse	M	CD45.1	1:100	Clone A20, cat no. 110702; Biolegend, CA, USA
6) Mouse	M	CD45.2-PE-Cy7	1:200	Clone 104; BD Biosciences, VIC, Australia
7) Rabbit	M, R	Caspase-1	1:100	Cat. no. AB_2068895; Santa Cruz Biotechnology, TX, USA
8) Mouse	H	Caspase-1	0.1 µg/ml	Cat. no. sc56036; Santa Cruz Biotechnology, TX, USA
9) Rabbit	H, M	ASC	1 µg/ml	Clone AL177; AdipoGen, CA, USA
10) Rabbit	H	NLRP3	1 µg/ml	Clone D2P5E; Cell Signaling, CA, USA
11) Mouse	N. A.	FLAG	1 µg/ml	Cat. no. F1804; Sigma-Aldrich, NSW, Australia
12) Rabbit	H, M, R	Tubulin	0.9 ng/ml	Cat. no. F1804600-401-880; Rockland, PA, USA
13) Rat	M	E-cadherin	5 µg/ml	Clone DECMA-1; Abcam, MA, USA
14) Rabbit	H, M, R	NOD1	1:100	Cat. no. 3545; Cell Signaling Technology, MA, USA
15) Rabbit	R	Alexa Fluor®488	1:400	Cat. no. A-21210; Thermo Fisher Scientific, VIC, AUS
16) Goat	Rabbit	Alexa Fluor®594	1:400	Cat. no. A-11012; Thermo Fisher Scientific, VIC, AUS
17) Goat	Rabbit	Alex Fluor®800	0.33-0.67 ng/ml	Cat. no. A32735; Thermo Fisher Scientific, VIC, AUS

Validation

All commercial antibodies in this study were validated, based on the manufacturers' websites. Antibodies were used for the appropriate animal host and application(s), as per the information provided on those websites. For each antibody, we have provided in Supplementary Table 1 the host species source, species reactivity, catalogue numbers and dilutions used.
The anti-H. pylori antibody was produced "in-house" and previously validated in the work of Ferrero, R. L. et al. "Recombinant antigens prepared from the urease subunits of Helicobacter spp.: Evidence of protection in a mouse model of gastric infection." Infect. Immun. 62, 4981-4989 (1994).
Further information for each commercial antibody used in the study is provided below.

1) Rat anti-IL-18: https://www.rndsystems.com/products/mouse-il-18-il-1f4-antibody-74_d047-3
Nussbaumer, O., et al., Blood 118, 2743-2751 (2011). Tu, A., et al., J. Exp. Med. 205, 233-244 (2008). Wu, C., J. Immunol. 170, 5571-5577 (2003). Sugawara, S., J. Immunol. 167, 6568-6575 (2001). Dao, T., et al., Cell Immunol. 173, 230-235 (1996). Micallef, M., et al., Eur. J. Immunol. 26, 1647-1651 (1996). Ushio, S., et al., J. Immunol. 156, 4274-4279 (1996). Okamura, H., et al., Nature 378, 88-91 (1995)

2) Rat anti-IL-18: https://www.citeab.com/antibodies/800749-sc-7954-il-18-h-173
El-Mezzein, R.E., et al. 2001. Increased secretion of IL-18 in vitro by peripheral blood mononuclear cells of patients with bronchial asthma and atopic dermatitis. Clin. Exp. Immunol. 126: 193-198. AbuElhija, M., et al. 2008. Lipopolysaccharide increased the expression levels of IL-18, ICE and IL-18 R in murine Leydig cells. Am. J. Reprod. Immunol. 60: 151-159. Abu Elhija, M., et al. 2008. LPS increases the expression levels of IL-18, ICE and IL-18 R in mouse testes. Am. J. Reprod. Immunol. 60: 361-371. Ojala, J., et al. 2009. Expression of interleukin-18 is increased in the brains of Alzheimer's disease patients. Neurobiol. Aging 30: 198-209. Tsai, P.Y., et al. 2011. Epigallocatechin-3-gallate prevents lupus nephritis development in mice via enhancing the Nrf2 antioxidant pathway and inhibiting NLRP3 inflammasome activation. Free Radic. Biol. Med. 51: 744-754. Liu, D., et al. 2014. Activation of the Nlrp3 inflammasome by mitochondrial reactive oxygen species: a novel mechanism of albumin-induced tubulointerstitial inflammation. Int. J. Biochem. Cell Biol. 57: 7-19. Liu, X., et al. 2015. Remifentanil ameliorates liver ischemia-reperfusion injury through inhibition of interleukin-18 signaling. Transplantation 99: 2109-2117.

3) Rabbit anti-EpCam: https://www.abcam.com/products/primary-antibodies/epcam-antibody-ab71916.html
Zheng Y et al. PD-L1+CD8+ T cells enrichment in lung cancer exerted regulatory function and tumor-promoting tolerance. iScience 25:103785 (2022). Mass Cytometry, glucose transporter glut1 . PubMed: 35146396. Byers C et al. Genetic control of the pluripotency epigenome determines differentiation bias in mouse embryonic stem cells. EMBO J 41:e109445 (2022). PubMed: 34931323. Li Z et al. ESR1 mutant breast cancers show elevated basal cytokeratins and immune activation. Nat Commun 13:2011 (2022). PubMed: 35440136. Lei Z et al. EpCAM Is Essential to Maintaining the Immune Homeostasis of Intestines via Keeping the Expression of pIgR in the Intestinal Epithelium of Mice. Front Immunol 13:843378 (2022). PubMed: 35493520. Han S et al. Anti-Cancer Effects of YAP Inhibitor (CA3) in Combination with Sorafenib against Hepatocellular Carcinoma (HCC) in Patient-Derived Multicellular Tumor Spheroid Models (MCTS). Cancers (Basel) 14:N/A (2022). PubMed: 35681712

4) Rat anti-EpCam-APC: https://www.thermofisher.com/antibody/product/CD326-EpCAM-Antibody-clone-G8-8-Monoclonal/14-5791-81

Ferreirinha, P et al. Identification of fibroblast progenitors in the developing mouse thymus. Development 149:dev200513 (2022).PMID: 35587733 DOI: 10.1242/dev.200513. Goga A et al. miR-802 regulates Paneth cell function and enterocyte differentiation in the mouse small intestine. Nat Commun 12:3339 (2021). PMID: 34099655 doi: 10.1038/s41467-021-23298-3.

5) Anti-CD45.1: https://www.biolegend.com/en-us/products/purified-anti-mouse-cd45-1-antibody-200?GroupID=BLG1933

Zhang S, et al. 2017. Nature.. 10.1038/nature24283. Prado C, et al. 2021. J Neuroinflammation. 18:292. Orecchioni M, et al. 2022. Methods Mol Biol. 2419:779. Hosoi A, et al. 2008. Cancer Res. 68:3941. Gunawan M, et al. 2017. Sci Rep. . 10.1038/s41598-017-16999-7. Matsuba S, et al. 2017. Front Immunol. . 10.3389/fimmu.2017.01538. De Simone G, et al. 2021. Immunity. Kenna T, et al. 2008. Blood. 111:2091. Oyarce K, et al. 2018. Front Immunol. 9:112. Guilliams M, et al. 2022. Cell. 185:379. Galbas T, et al. 2017. J Immunol. 198(2):852-861. Lee-Chang C, et al. 2021. J Exp Med. 218:. PubMed

6) Mouse anti-CD45.2-PE-Cy7: https://www.bdbiosciences.com/en-au/products/reagents/flow-cytometry-reagents/research-reagents/single-color-antibodies-ruo/pe-mouse-anti-mouse-cd45-2.560695

Greimers R et al. Improved four-color flow cytometry method using fluo-3 and triple immunofluorescence for analysis of intracellular calcium ion ([Ca2+]i) fluxes among mouse lymph node B- and T-lymphocyte subsets. Cytometry. 1996; 23(3):205-217. (Methodology: Flow cytometry). Johnson P et al. Identification of the alternatively spliced exons of murine CD45 (T200) required for reactivity with B220 and other T200-restricted antibodies. J Exp Med. 1989; 169(3):1179-1184. (Biology). Morse HC et al. Genetic nomenclature for loci controlling mouse lymphocyte antigens. Immunogenetics. 1987; 25(2):71-78. (Biology).

7) Rabbit anti-caspase-1: https://antibodyregistry.org/search.php?q=AB_2068895

PMID:26968342, PMID:27863209, PMID:28270571, PMID:28666573, PMID:29033131, PMID:29290574, PMID:29414684, PMID:29447697, PMID:29681442, PMID:30392956, PMID:30404007, PMID:30445425, PMID:30485804, PMID:30770245, PMID:32096759

8) Mouse anti-caspase-1: https://www.scbt.com/p/caspase-1-antibody-14f468

The prodomain of caspase-1 enhances Fas-mediated apoptosis through facilitation of caspase-8 activation. | Tatsuta, T., et al. 2000. J Biol Chem. 275: 14248-54. PMID: 10799503. Caspase pathways, neuronal apoptosis, and CNS injury. | Eldadah, BA. and Faden, AI. 2000. J Neurotrauma. 17: 811-29. PMID: 11063050. Proteomic analysis of mechanisms of hypoxia-induced apoptosis in trophoblastic cells. | Ishioka, S., et al. 2006. Int J Med Sci. 4: 36-44. PMID: 17299580. NF-kappaB activation by the Toll-IL-1 receptor domain protein MyD88 adapter-like is regulated by caspase-1. | Miggin, SM., et al. 2007. Proc Natl Acad Sci U S A. 104: 3372-7. PMID: 17360653. Anthrax lethal toxin kills macrophages in a strain-specific manner by apoptosis or caspase-1-mediated necrosis. | Muehlbauer, SM., et al. 2007. Cell Cycle. 6: 758-66. PMID: 17374996. Targeting caspase-1 in sepsis: a novel approach to an old problem. | Matute-Bello, G. 2007. Intensive Care Med. 33: 755-757. PMID: 17384934. Interleukin-1 beta converting enzyme requires oligomerization for activity of processed forms in vivo. | Gu, Y., et al. 1995. EMBO J. 14: 1923-31. PMID: 7743999

9) Rabbit anti-ASC: https://adipogen.com/ag-25b-0006-anti-asc-pab-al177.html

NALP3 forms an IL-1beta-processing inflammasome with increased activity in Muckle-Wells autoinflammatory disorder: L. Agostini, et al.; Immunity 20, 319 (2004). P2X7 Receptor Differentially Couples to Distinct Release Pathways for IL-1beta in Mouse Macrophage: P. Pelegrin, et al.; J. Immunol. 180, 7147 (2008). Inflammatory role of ASC in antigen-induced arthritis is independent of caspase-1, NALP-3, and IPAF: L. Kolly, et al.; J. Immunol. 183, 4003 (2009). Activation of autophagy by inflammatory signals limits IL-1b production by targeting ubiquitinated inflamma- somes for destruction: C.-S. Shi, et al.; Nat. Immunol. 13, 255 (2012). NLRP3 is activated in Alzheimer's disease and contributes to pathology in APP/PS1 mice: M.T. Heneka, et al.; Nature 493, 674 (2013)

10) Rabbit anti-NLRP3: https://www.cellsignal.com/products/primary-antibodies/nlrp3-d2p5e-rabbit-mab/13158

Elinav, E. et al. (2011) Immunity 34, 665-79. Inohara, N. et al. (1999) J Biol Chem 274, 14560-7. Ogura, Y. et al. (2001) J Biol Chem 276, 4812-8. Sabbah, A. et al. (2009) Nat Immunol 10, 1073-80. Mariathasan, S. et al. (2004) Nature 430, 213-8. Agostini, L. et al. (2004) Immunity 20, 319-25. Martinon, F. et al. (2002) Mol Cell 10, 417-26. Franchi, L. et al. (2012) Nat Immunol 13, 325-32. Guarda, G. et al. (2011) J Immunol 186, 2529-34.

11) Rabbit anti-FLAG: https://www.sigmaaldrich.com/AU/en/product/sigma/f1804

Roquin binds microRNA-146a and Argonaute2 to regulate microRNA homeostasis.

Monika Srivastava et al.Nature communications, 6, 6253-6253 (2015-02-24)

Hepatitis C Virus Infection Induces Hepatic Expression of NF-κB-Inducing Kinase and Lipogenesis by Downregulating miR-122. Brianna Lowey et al. mBio, 10(4) (2019-08-01)

12) Rabbit anti-tubulin: https://www.rockland.com/categories/primary-antibodies/alpha-tubulin-antibody-600-401-880/

Gerlach, P et al. Structure and regulation of the nuclear exosome targeting complex guides RNA substrates to the exosome. Molecular Cell (2022) [Applications WB, IB, PCA]. Dillinger, AE et al. CCN2/CTGF-A Modulator of the Optic Nerve Head Astrocyte. Frontiers in Cell and Developmental Biology (2022) [Applications WB, IB, PCA]. Wang-Eckhardt, L et al. Absence of endogenous carnosine synthesis does not increase protein carbonylation and advanced lipoxidation end products in brain, kidney or muscle. Amino Acids (2022) [Applications WB, IB, PCA]

13) Rat anti-E-cadherin: https://www.sigmaaldrich.com/AU/en/product/mm/mabt26

Kupffer Cell-Derived Tnf Triggers Cholangiocellular Tumorigenesis through JNK due to Chronic Mitochondrial Dysfunction and ROS. Detian Yuan et al. Cancer cell, 31(6), 771-789 (2017-06-14). Functional roles for PIEZO1 and PIEZO2 in urothelial mechanotransduction and lower urinary tract interoception. Marianela G Dalghi et al. JCI insight, 6(19) (2021-09-01). Effect of CUL4A on the metastatic potential of lung adenocarcinoma to the bone.

Pei-Pei Cao et al. Oncology reports, 43(2), 662-670 (2020-01-03)

14) Rabbit anti-NOD1: https://www.cellsignal.com/products/primary-antibodies/nod1-antibody/3545

Inohara, N. et al. (1999) J. Biol. Chem. 274, 14560-14567. Inohara, N. and Nuñez, G. (2003) Nat. Rev. Immunol. 3, 371-382. Fritz, J.H. et al. (2006) Nat .Immunol. 7, 1250-1257. Girardin, S.E. et al. (2001) EMBO Rep. 2, 736-742. Inohara, N. et al. (2001) J. Biol. Chem. 276, 2551-2554. Inohara, N. et al. (2000) J. Biol. Chem. 275, 27823-27831. Hysi, P. et al. (2005) Hum. Mol. Genet. 14, 935-941. McGovern, D.P. et al. (2005) Hum. Mol. Genet. 14, 1245-1250.

15) Anti-rat Alexa®488: https://www.thermofisher.com/antibody/product/Rabbit-anti-Rat-IgG-H-L-Cross-Adsorbed-Secondary-Antibody-Polyclonal/A-21210
Increased Dystrophin Production With Golodirsen in Patients With Duchenne Muscular Dystrophy. Neurology (2023). Serum inflammatory cytokines as disease biomarkers in the DE50-MD dog model of Duchenne muscular dystrophy. Dis Model Mech (2022). Alveolar epithelial progenitor cells drive lung regeneration via dynamic changes in chromatin topology modulated by lineage-specific Nkx2-1 activity bioRxiv (2022). A nanodomain-anchored scaffolding complex is required for the function and localization of phosphatidylinositol 4-kinase alpha in plants. Plant Cell (2022). ITGB6 inhibits the proliferation of porcine skeletal muscle satellite cells. Cell Biol Int (2022)

16) Anti-rabbit Alexa®594: https://www.thermofisher.com/antibody/product/Goat-anti-Rabbit-IgG-H-L-Cross-Adsorbed-Secondary-Antibody-Polyclonal/A-11012
New botulinum neurotoxin constructs for treatment of chronic pain. Life Sci Alliance (2023). Targeting an essential Plasmodium cold shock protein to block growth and transmission of malaria parasite. iScience (2023). Pan-sarbecovirus prophylaxis with human anti-ACE2 monoclonal antibodies. Nat Microbiol (2023). Polypyrimidine tract binding protein knockdown reverses depression-like behaviors and cognition impairment in mice with lesioned cholinergic neurons. Front Aging Neurosci (2023). Circadian glucocorticoids throughout development. Front Neurosci (2023).

17) Anti-rabbit Alex Fluor® 800: https://www.thermofisher.com/antibody/product/Goat-anti-Rabbit-IgG-H-L-Highly-Cross-Adsorbed-Secondary-Antibody-Polyclonal/A32735
Endothelial deletion of PTBP1 disrupts ventricular chamber development. Nat Commun (2023). DNMT3A low-expression is correlated to poor prognosis in childhood B-ALL and confers resistance to daunorubicin on leukemic cells. BMC Cancer (2023). Characterization of huntingtin interactomes and their dynamic responses in living cells by proximity proteomics. J Neurochem (2023). An antisense amido-bridged nucleic acid gapmer oligonucleotide targeting SRRM4 alters REST splicing and exhibits anti-tumor effects in small cell lung cancer and prostate cancer cells. Cancer Cell Int (2023). FMRP activity and control of Csw/SHP2 translation regulate MAPK-dependent synaptic transmission. PLoS Biol (2023).

Eukaryotic cell lines

Policy information about cell lines and Sex and Gender in Research

Cell line source(s)

AGS gastric cancer cells stably expressing shRNA to either EGFP or NOD1 (generated in-house). AGS control (Cas9) or NOD1 knockout cells generated by CRISPR/Cas9 gene editing (generated in-house). All four cell lines have been published previously - Viala et al. (2004) "Nod1 responds to peptidoglycan delivered by the Helicobacter pylori cag pathogenicity island." Nature Immunol. 5, 1166-1174; Kaparakis et al. (2010) "Bacterial membrane vesicles deliver peptidoglycan to NOD1 in epithelial cells." Cell Microbiol. 12, 372-385. Grubman et al. (2010) "The innate immune molecule, NOD1, regulates direct killing of Helicobacter pylori by antimicrobial peptides." Cell Microbiol. 12, 626-639; 71. Tran et al. (2018) "NOD1 is required for Helicobacter pylori induction of IL-33 responses in gastric epithelial cells." Cell Microbiol. 20, e12826.)
The mouse GSM06 GEC line (Riken Cell Bank RCB1779) used in our study Ferrero et al. (2008) "NF-kB activation during acute Helicobacter pylori infection in mice. Infect. Immun. 76, 551-561. "
HEK-Blue™ IL-18 reporter cell line (InvivoGen, San Diego, CA, USA).
Organoids from: Nod1+/+ and Nod1-/- mice.
Primary gastric epithelial cells from: wild-type, Casp1-/-, Nlrp3-/-, Nod1-/-, Pycard-/- and Ripk2-/- mice.
Bone marrow-derived macrophages from: wild-type, Nlrp3-/-, Pycard-/-, Nod1fl/fl and Nod1fl/fl x lysM-cre mice.
Primary cells were generated from both male and female mice.

Authentication

AGS stable knockdowns and knockout cells were verified by qPCR or PCR but, otherwise, the cell lines were not authenticated.

Mycoplasma contamination

All cell lines were routinely tested for mycoplasma contamination and tested negative.

Commonly misidentified lines
(See ICLAC register)

No commonly used strains were included in the study.

Animals and other research organisms

Policy information about studies involving animals; ARRIVE guidelines recommended for reporting animal research, and Sex and Gender in Research

Laboratory animals	Wild-type, Casp1-/-, Il18-/-, Nlrp1-/-, Nlrp1+/-, Nlrp3-/-, Nlrc4-/-, Nod1-/-, Nod1fl/fl, Nod1fl/fl x LysM-Cre mice and Pycard-/- mice on the C57BL/6J genetic background. Mice were matched according to sex and age (6-8 weeks for infection studies and 3-4 weeks for the generation of primary gastric epithelial cells/organoids).<br>Mice were housed in SPF (specific pathogen free) facilities with controlled temperature (18–22°C) and humidity (50–60%) and a 12-h dark/12-h light cycle. These conditions are now reported in the manuscript.
Wild animals	Not applicable
Reporting on sex	As there were no known sex-based differences for the phenotypes under investigation in the study, and to reduce the numbers of mice having to be bred (and culled), all experiments used animals of both sexes. The lack of sex-basis was confirmed in preliminary experiments.
Field-collected samples	No wild animals were used in the study.
Ethics oversight	All animal procedures were approved by the Animal Ethics Committees at WEHI (2014.004, 2011.014, 2008.022) and Monash Medical Centre (Monash University; MMCA/2015/43).

Note that full information on the approval of the study protocol must also be provided in the manuscript.

Flow Cytometry

Plots

Confirm that:

- [x] The axis labels state the marker and fluorochrome used (e.g. CD4-FITC).
- [x] The axis scales are clearly visible. Include numbers along axes only for bottom left plot of group (a 'group' is an analysis of identical markers).
- [x] All plots are contour plots with outliers or pseudocolor plots.
- [x] A numerical value for number of cells or percentage (with statistics) is provided.

Methodology

Sample preparation	Not applicable
Instrument	FACS Aria Fusion (Beckman Coulter)
Software	FlowJo™ (vers. 10.5)
Cell population abundance	Single-cell suspensions were prepared from mouse stomachs in 2% FCS in Hank's Balanced Salt Solution without calcium and magnesium and 5 mM EDTA, then strained through 70 μm cell strainers. The remaining pieces of tissues were further digested at 37˚C in 2% FCS in RPMI, containing 1 mg/ml collagenase Type 1, 0.4 units Dispase and 0.01 mg/ml DNase.<br>AGS cells were detached from culture plates by the addition of 0.25% Trypsin and 1 mM EDTA.
Gating strategy	Cells were gated according to the expression of epithelial (EpCam) or immune cell (CD45) markers. Alternatively, cells were gated for annexin/PI staining.

- [x] Tick this box to confirm that a figure exemplifying the gating strategy is provided in the Supplementary Information.
